# Decoding Single Cell Morphology in Osteotropic Breast Cancer Cells for Dissecting Their Migratory, Molecular and Biophysical Heterogeneity

**DOI:** 10.3390/cancers14030603

**Published:** 2022-01-25

**Authors:** Lila Bemmerlein, Ilker A. Deniz, Jana Karbanová, Angela Jacobi, Stephan Drukewitz, Theresa Link, Andy Göbel, Lisa Sevenich, Anna V. Taubenberger, Pauline Wimberger, Jan Dominik Kuhlmann, Denis Corbeil

**Affiliations:** 1 Department of Gynecology and Obstetrics, Medical Faculty and University Hospital Carl Gustav Carus, Technische Universität Dresden, 01307 Dresden, Germany; lila.bemmerlein@uniklinikum-dresden.de (L.B.); theresa.link@uniklinikum-dresden.de (T.L.); Pauline.Wimberger@uniklinikum-dresden.de (P.W.); 2 National Center for Tumor Diseases (NCT), 01307 Dresden, Germany; German Cancer Research Center (DKFZ), 69120 Heidelberg, Germany; Faculty of Medicine, University Hospital Carl Gustav Carus, Technische Universität Dresden, 01307 Dresden, Germany; Helmholtz-Zentrum Dresden-Rossendorf (HZDR), 01307 Dresden, Germany; stephan.drukewitz@uniklinikum-dresden.de; 3 German Cancer Consortium (DKTK), Partner Site Dresden and German Cancer Research Center (DKFZ), 69120 Heidelberg, Germany; 4 Biotechnology Center (BIOTEC) and Center for Molecular and Cellular Bioengineering (CMCB), Technische Universität Dresden, 01307 Dresden, Germany; ilker_ali.deniz@tu-dresden.de (I.A.D.); jana.karbanova@tu-dresden.de (J.K.); angela.berg-jacobi@tu-dresden.de (A.J.); anna.taubenberger@tu-dresden.de (A.V.T.); 5 Max Planck Institute for the Science of Light and Max-Planck-Zentrum für Physik und Medizin, 91058 Erlangen, Germany; 6 Core Unit for Molecular Tumor Diagnostics (CMTD), National Center for Tumor Diseases (NCT), Partner Site Dresden, German Cancer Consortium (DKTK), Dresden, German Cancer Research Center (DKFZ), 69120 Heidelberg, Germany; 7 Department of Medicine III and Center for Healthy Aging, Technische Universität Dresden, 01307 Dresden, Germany; andy.goebel@uniklinikum-dresden.de; 8 German Cancer Consortium (DKTK), Partner Site Frankfurt/Mainz and German Cancer Research Center (DKFZ), 69120 Heidelberg, Germany; sevenich@gsh.uni-frankfurt.de; 9 Institute for Tumor Biology and Experimental Therapy, Georg-Speyer-Haus, 60596 Frankfurt am Main, Germany; 10 Frankfurt Cancer Institute (FCI), Goethe University Frankfurt, 60594 Frankfurt am Main, Germany

**Keywords:** bone metastasis, atomic force microscopy, breast cancer cell, cell morphology, lectin, migration, membrane protrusion, transcriptome

## Abstract

**Simple Summary:**

Predicting metastatic bone tropism in breast cancer by simply analyzing cell morphology would be highly desirable, in order to identify breast cancer patients at high risk for bone metastasis or to develop innovative bone-targeting drugs. In the current in vitro study, we show that MDA-MB-231 breast cancer cells are morphologically heterogeneous and exhibit a wide range of stable morphologies on a single cell level. Using the highly osteotropic derivative cell lines of MDA-MB-231 (MDA-MET, MDA-BONE), we demonstrate that single cell morphology reflects the molecular, migratory and biophysical nature of a given breast cancer cell and is specifically altered upon adoption of a bone-tropic phenotype. Thus, we propose that cell morphology could be an informative readout for understanding breast cancer heterogeneity and for predicting bone-metastases in breast cancer patients.

**Abstract:**

Breast cancer is a heterogeneous disease and the mechanistic framework for differential osteotropism among intrinsic breast cancer subtypes is unknown. Hypothesizing that cell morphology could be an integrated readout for the functional state of a cancer cell, we established a catalogue of the migratory, molecular and biophysical traits of MDA-MB-231 breast cancer cells, compared it with two enhanced bone-seeking derivative cell lines and integrated these findings with single cell morphology profiles. Such knowledge could be essential for predicting metastatic capacities in breast cancer. High-resolution microscopy revealed a heterogeneous and specific spectrum of single cell morphologies in bone-seeking cells, which correlated with differential migration and stiffness. While parental MDA-MB-231 cells showed long and dynamic membrane protrusions and were enriched in motile cells with continuous and mesenchymal cell migration, bone-seeking cells appeared with discontinuous mesenchymal or amoeboid-like migration. Although non-responsive to CXCL12, bone-seeking cells responded to epidermal growth factor with a morphotype shift and differential expression of genes controlling cell shape and directional migration. Hence, single cell morphology encodes the molecular, migratory and biophysical architecture of breast cancer cells and is specifically altered among osteotropic phenotypes. Quantitative morpho-profiling could aid in dissecting breast cancer heterogeneity and in refining clinically relevant intrinsic breast cancer subtypes.

## 1. Introduction

Breast cancer is the most common malignancy in females worldwide [1]. Due to early detection and innovative treatment, the prognosis of breast cancer patients is generally favorable. Nevertheless, about 20–30% of patients develop distant metastases over time, resulting in poor overall survival [2,3]. The occurrence of bone metastasis is a severe complication in breast cancer and particularly osteolytic bone metastases often have a detrimental impact on quality of life and survival, e.g., by inducing pathological fractures, nerve compression syndromes or severe pain [4,5]. Loco-regional treatment of bone metastasis and current bone targeting agents, such as anti-resorptive drugs or bone-seeking radiopharmaceuticals, are often ineffective, so that bone-metastatic breast cancer is essentially incurable [6].

The metastatic cascade of breast cancer cells comprises five key steps: (1) acquisition of an invasive phenotype; (2) entrance of tumor cells into the blood vessels (intravasation); (3) hematogenous spread; (4) extravasation; and (5) colonization at a distant metastatic site [7]. Interestingly, breast cancer subtypes are associated with the diverging tropism of metastatic spread in humans, as, for example, hormone receptor (HR)-positive breast cancer preferably metastasizes to the bone, whereas human epidermal growth factor 2 (HER2)-positive or triple-negative (HR-/HER2-) breast cancers more probably spread to visceral organs [2,8]. The fact that certain tumor subtypes have a metastatic predilection to specific organs, referred to as ‘seed and soil hypothesis’, has been recognized since 1889 and indicates that the organ preference of tumor metastasis is the final result of a favorable interaction between metastatic tumor cells (the ‘seed’) and the organ environment (the ‘soil’) [9]. Up to now, several molecular mechanisms have been proposed to direct the organ tropism of metastatic tumor cells, such as the hijacking of physiological cellular migration routes, mediated by homeostatic chemokines and their cognate receptors [10]. However, the exact cellular and microenvironmental mechanisms determining organ tropism of breast cancer cells, particularly their ability to colonize the bone, remain to be elucidated. From a clinical–translational point of view, a deeper knowledge of the molecular routes of bone-metastasis is highly desirable, in order to identify early breast cancer patients with high risk of bone-relapse and to define innovative therapeutic targets for bone-metastatic breast cancer.

Human triple-negative MDA-MB-231 (MB-231) breast cancer cells have been used in many metastasis models in mice and exhibit a heterogeneous spectrum of organ tropism, including lung, bone, brain, ovary or adrenal glands [11,12,13,14,15]. By repeated in vivo selection in mice, two highly metastatic bone-seeking variants have been established from the parental MB-231, referred to as MDA-MB-231-MET (MET) and MDA-MB-231-BONE (BONE) [11,16]. These derivative cell lines exclusively induce bone-metastases in mice, shaped by large osteolytic lesions and are therefore frequently used for in vivo models of bone metastasis [17]. Moreover, it was revealed that the bone seeking MET and BONE cells show molecular differences compared to parental MB-231 on the level of gene expression [16], glycosylation profile [18] or responsiveness to the cytokine TGF-β [11]. Interestingly, a recent study proposed that the single cell morphology of MB-231 cells in vitro is an integrated outcome of the molecular and biophysical properties of breast cancer cells and encodes their functional state, such as their metastatic potential [13]. However, as this study focused exclusively on lung metastasis, the in vitro spectrum of morphotypes in exclusively osteotropic breast cancer cells and its relation to their functional traits is completely unknown.

Therefore, the objective of the current study was (i) to establish a comprehensive catalogue of the migratory, molecular and biophysical traits of MDA-MB-231 breast cancer cells; (ii) to compare it to their enhanced bone-seeking derivatives MET and BONE; and (iii) to integrate these findings with single cell morphology profiles.

## 2. Materials and Methods

### 2.1. Reagents

CXCL12 chemokine was obtained from Abcam (catalog number #ab9798, Cambridge, UK) and the epidermal growth factor (EGF) from PeproTech Inc. (#AF-100-15, Cranbury, NJ, USA). CXCL12 was resuspended in 0.1% bovine serum albumin (BSA) solution at a final concentration of 10 ng/µL, while EGF was resuspended in sterile water at a final concentration of 10 ng/µL. Alexa Fluor^®^488-conjugated Phalloidin was purchased from Thermo Fisher Scientific (Waltham, MA, USA). The 4,6-diamidino-2-phenylindole (DAPI) was purchased from Sigma Aldrich (#32670, Darmstadt, Germany).

### 2.2. Antibodies

The primary and secondary antibodies used in this study are presented in Tables S1 and S2, respectively.

### 2.3. Cell Culture

The human triple-negative breast cancer cell line, MDA-MB-231 (MB-231; ATCC, Manassas, VA, USA) and its bone-seeking sub-lines, MDA-MB-231-MET (MET) [16] and MDA-MB-231-BONE (BONE) [11], were used as described [19,20]. MET cells were kindly provided by Dr. Larry J. Suva (Center for Orthopedic Research, University of Arkansas, Little Rock, AR, USA), while BONE cells were obtained from the University of Texas (San Antonio, TX, USA). Their intrinsic subtype was re-analyzed by immunohistochemistry for estrogen receptor (ER), progesterone receptor (PR), HER2 and Ki67 at the Institute of Pathology (Medical Faculty, TU Dresden). They were confirmed to be triple negative and had a high proliferative activity. All three cell lines were cultured in a 1:1 mix of Dulbecco’s modified Eagle’s medium and Ham’s F-12 nutrient mixture (DMEM:F12; #11320074, Gibco Corp., Carlsbad, CA, USA) supplemented with 10% fetal bovine serum (FBS, #F7524, Lot BCCB7352, Sigma Aldrich) and 100 U/mL penicillin and 100 μg/mL streptomycin (P/S, Gibco Corp.) at 37 °C in a 5% CO_2_ humidified incubator. Alternatively, they were maintained in Roswell Park Memorial Institute Medium (RPMI)-1640 (#21875034, Gibco Corp.) supplemented with 10% FBS and P/S. The brain-seeking variant MDA-MB-231-BrM2 (BRAIN) was kindly provided by Dr. Joan Massagué (Memorial Sloan Kettering Cancer Center, New York, NY, USA) [21]. They were cultivated in DMEM medium (#61965026, Gibco Corp.) supplemented with 10% FBS and P/S at 37 °C under 5% CO_2_ atmosphere. Cells were regularly tested for mycoplasma contamination by polymerase chain reaction at the Institute for Microbiology (Medical Faculty, TU Dresden).

### 2.4. Time-Lapse Video Microscopy

Cells were seeded on fibronectin-coated glass-bottom 35-mm dishes (#P35G-1.5-14-C, MatTek Corp., Ashland, MA, USA) and cultured under subconfluence. Before imaging, the medium was replaced with a fresh one. In some experiments, CXCL12 (100 ng/mL) or EGF (25 ng/mL) or both were added. During the time-lapse recording, cells were kept in a 37 °C chamber with 5% CO_2_ atmosphere. Serial phase-contrast images were captured with an inverted microscope (Zeiss Axiovert 200M microscope, 20×/0.5 Ph2 objective, Wetzlar, Germany) equipped with a Hamamatsu Orca Flash 4.0 sCMOS camera (Hamamatsu Photonics, Hamamatsu City, Japan). The images were acquired at 5-min intervals for a period of 12 h. All images were processed with Fiji software [22]. Tracking of cells were performed with the Trackmate plugin [23].

### 2.5. Fluorescence Labeling, Lectin Cytochemistry and Laser Scanning Confocal Microscopy

Breast cancer cells growing on fibronectin-coated glass coverslips (50 µg/mL) were cell-surface labeled for various protein markers using specific primary antibodies and appropriate fluorochrome-coupled secondary antibodies (Tables S1 and S2) or probed with rhodamine-coupled Wheat Germ Agglutinin (WGA) (#RL-1022, Vector Laboratories, Burlingame, CA, USA) or various fluorescein isothiocyanate (FITC)-coupled lectins obtained from Lectin kits I-III, Fluorescein (#FLK-2100, FLK-3100, FLK-4100, respectively) and Sambucus Nigra Lectin (SNA), Fluorescein (FL-1301-2), all from Vector Laboratories. After washing with PBS and then ice-cold Ca/Mg^-^PBS (PBS containing 1 mM CaCl_2_ and 0.5 mM MgCl_2_), cells were incubated in ice-cold blocking buffer I (Ca/Mg-PBS containing 0.2% gelatin) for 15 min at 4 °C. Primary antibodies or lectins diluted in blocking buffer I were added for 30 min at 4 °C. After washing with ice-cold Ca/Mg-PBS and PBS, labeled cells were fixed with 4% paraformaldehyde (PFA) for 30 min at room temperature, and then quenched with 50 mM NH_4_Cl for 10 min. For the indirect immunofluorescence, cells were further incubated in blocking buffer II (PBS containing 0.2% gelatin) for 20 min, and incubated with fluorochrome-conjugated secondary antibody (1:600; Molecular Probes, Life Technologies, Carlsbad, CA, USA) diluted in blocking buffer II for 30 min at room temperature. Nuclei were stained with DAPI (1 μg/mL). Samples were subsequently washed in PBS and H_2_O and mounted in Mowiol^®^4-88 (Merck, Darmstadt, Germany). Alternatively, after cell-surface labeling, PFA-fixed cells were permeabilized for 30 min at room temperature with 0.2% saponin diluted in blocking buffer II, and then stained with Alexa Fluor^®^488-conjugated Phalloidin or immunolabeled for cytoplasmic proteins as above. Samples were observed with a Carl Zeiss Axiovert 200M Apotome microscope or a Zeiss LSM 700 laser scanning confocal microscope. Various Zeiss objectives were used (10×/0.3.Ph1, 20×/0.8 and 40×/1.3 oil and 63×/1.4 oil). The images acquired under the same setting for all cell lines were processed with Fiji and figures were prepared with Adobe Illustrator.

### 2.6. Scanning Electron Microscopy

Breast cancer cells growing on fibronectin-coated glass coverslips for 2 days were prepared for scanning electron microscopy (SEM) analysis as described previously [24]. Briefly, after 2 days, they were fixed in 2% glutaraldehyde for 1 h at room temperature and then overnight at 4 °C. Following 2-h post-fixation in 1% osmium tetroxide at 4 °C, they were subjected to dehydration in an acetone gradient (25–100%) and critical point-dried in a CO_2_ system (Critical Point Dryer, Leica Microsystems, EM CPD 300, Wetzlar, Germany). Samples were then sputter-coated with gold (sputter-coating device SCD 050; BAL-TEC GmbH, Witten, Germany) and examined at a 5-kV accelerating voltage with a field emission scanning electron microscope (Jeol JSM 7500F, Freising, Germany).

### 2.7. Atomic Force Microscopy Indentation

A total of 48 h prior to an atomic force microscopy (AFM) probing, cells were seeded into glass bottom dishes (WPI, Sarasota, FL, USA) and cultured as described above. For AFM indentation experiments, a Nanowizard 4 (JPK Instruments/Bruker, Berlin, Germany) was used. Arrow-TL1 cantilevers (Nanoworld, Neuchatel, Switzerland) with a nominal spring constant of 0.035–0.050 N/m that had been modified with a polystyrene bead of 5-µm diameter (Microparticles GmbH, Berlin, Germany) were calibrated by the thermal noise method, using built-in procedures of the AFM software. To probe a selected cell, the cantilever/bead was positioned over the nuclear region and three repeated indentation tests were performed using an approach/retraction speed of 5 µm/s and a relative set point of 2.5 nN. For morphotype classification, a phase-contrast image was recorded from each cell. All experiments were performed at 37 °C using a Petri dish heater (JPK instruments/Bruker) and in a CO_2_-independent medium (Gibco Corp.). The resulting force distance curves were analyzed using the JPK data processing software (JPK instruments/Bruker). Force distance data were corrected for the tip sample separation and fitted with the Hertz/Sneddon model fit for a spherical indenter to extract the apparent Young’s modulus [25,26,27]. A Poisson ratio of 0.5 was assumed.

### 2.8. Real-Time Deformability Cytometry

Subconfluent cells that had grown for 48 h were detached using TrypLE (Gibco Corp.), spun down by centrifugation at 180× *g* for 4 min, and resuspended in 0.5% methylcellulose/PBS containing 50 µg/mL DNAse (>2000U/mg protein, Sigma Aldrich). Real-time deformability cytometry (RT-DC) measurements were performed as described previously [28,29]. Briefly, about 30 µL of sample volume were loaded into a 1 mL syringe and pumped through a 30-µm narrow channel constriction in a microfluidic chip. A total flow rate of 0.16 µL/s (sample flow 0.04 µL/s, sheath flow 0.12 µL/s) was chosen. At the end of the microfluidic channel, images of cells were captured by a high-speed camera and the contour was automatically detected using shape-in software (Version 2.0.8, Zellmechanik Dresden, Dresden, Germany). From the contour, the cross-sectional area (*A*) and the deformation ($D=1-2\sqrt{\pi A}/l$; *l*—perimeter of the contour) were derived. Moreover, a brightfield image was acquired for every measured cell, making the data available for multiparametric offline analysis. Data analysis and calculation of the apparent elastic modulus was performed in ShapeOut 1.0.6 (available at https://github.com/ZELLMECHANIK-DRESDEN/ShapeOut (accessed on 28 April 2020)).

### 2.9. Flow Cytometry

Cells growing in Nunc^TM^ Petri dishes (Thermo Fisher Scientific) were harvested by trypsin/EDTA treatment for 3 min at 37 °C. After inactivation of trypsin, 2 washing steps with PBS and centrifugation (5 min at 300× *g*), cells were resuspended in PBS or Ca/Mg-PBS (for lectin labeling), containing 1% BSA and 100 μL-cell suspension aliquots were incubated with unconjugated or fluorochrome-conjugated primary antibodies (Table S1) or FITC- or biotin-conjugated lectins (see above, Lectin kit I, Biotinylated (BK-1000) and SNA, Biotinylated (B-1305-2), both from Vector Laboratories) for 30 min at 4 °C. When necessary, appropriate fluorochrome-conjugated secondary antibodies (Table S2) or allophycocyanin (APC)-conjugated streptavidin (1:200, BioLegend, San Diego, CA, USA) were applied for 30 min at 4 °C. After washing with PBS, 20,000 events were acquired on an LSRII flow cytometer (BD Biosciences, Franklin Lakes, NJ, USA). Instrument settings and gating strategies were established using the appropriate isotype antibody, secondary antibody, streptavidin or unstained controls. Data were analyzed using FlowJo software (FlowJo, LLC, Ashland, OR, USA). The median fluorescence intensity (MFI) was calculated as the difference between the MFI values obtained from the stained and negative controls (i.e., cell populations incubated with isotype primary antibody or secondary antibody alone in the case of CD markers, or with APC-conjugated streptavidin alone or without in the case of lectins).

### 2.10. Immunoblotting

Cells were harvested with a cell scraper in ice-cold PBS containing a Complete^TM^ protease inhibitor cocktail (Roche Diagnostics GmbH, Mannheim, Germany) and collected by centrifugation at 500× *g* for 5 min at 4 °C. The cell pellets were then solubilized with radio-immunoprecipitation assay buffer (RIPA buffer; 150 mM NaCl, 1% Nonidet P-40, 0.5% sodium deoxycholate, 0.1% sodium dodecyl sulfate (SDS), 50 mM Tris/HCl, pH 8) on ice for 30 min. After centrifugation (10 min, 16,000× *g*, 4 °C), the detergent lysates were removed, and an aliquot was used to determine the protein concentration using a Pierce^TM^ BCA Protein Assay Kit (#23225; Thermo Fisher Scientific). The samples were then stored at −20 °C until use. Samples were subjected to SDS-polyacrylamide-gel electrophoresis (SDS-PAGE; 7.5 or 12%) and transferred to polyvinylidene difluoride membranes (pore size: 0.45 µm; Millipore Corp., Belford, MA, USA) using a semi-dry transfer cell system (Cti, Idstein, Germany). After transfer, membranes were incubated either 1 h at room temperature or overnight at 4 °C in blocking buffer III (PBS containing 0.3% Tween-20 and 5% low fat milk powder). Afterward, membranes were incubated with primary antibody diluted in blocking buffer III for 1 h at room temperature. In all cases, antigen–antibody complexes were revealed using the appropriate horseradish peroxidase-conjugated secondary antibody (Jackson ImmunoResearch Europe Ltd., Ely, UK) (Table S2) followed by chemiluminescence detection (ECL System: GE Healthcare Life Sciences, Chicago, IL, USA). Membranes were exposed to Amersham Hyperfilm ECL (GE Healthcare Life Sciences), and developed using Optimax Mammo X-Ray film processor (Protec, Oberstenfeld, Germany). When necessary, Fiji software was used for the quantification of immunoblotted materials.

### 2.11. Transcriptome

For RNA-sequencing, cells were harvested after reaching 40% confluency and RNA extraction was performed by using the miRNeasy Mini Kit (Qiagen, Hilden, Germany) according to the manufacturer’s instructions. RNA libraries were prepared using TruSeq Stranded mRNA Library Prep Kit (Illumina, San Diego, CA, USA) from 1 μg total RNA. All barcoded libraries were pooled and sequenced on an Illumina NextSeq500 platform to obtain a minimum of 10 million reads per sample. All experiments were performed as triplicates for each cell line.

### 2.12. RNA-Seq Data Analysis

Raw reads from the sequencing data were trimmed using trimmomatic [30] and aligned using STAR [31]. Read counts were extracted from the alignments using the featureCounts method of the Rsubread package [32]. Differential gene expression analysis was performed using the DESeq2 R package [33]. All differentially expressed genes were determined by absolute log2 fold change >2 with a *q*-value of <0.01. Over-representation analysis of the differentially expressed genes in the Kyoto Encyclopedia of Genes and Genomes (KEGG) pathways were performed by the clusterProfiler R package [34]. The gene lists and pathway analyses were obtained from KEGG or Molecular Signatures Databases and protein interaction networks were generated by the Cytoscape plugin ClueGO [35]. Heatmaps were generated using the Pheatmap R package using log2 transformed normalized counts generated by DESeq2. To highlight the additional genes and show the overall expression levels, heatmaps were also prepared from raw expression data without differential expression filtering. To further validate the gene expression signature of the MB-231 cells with publicly available datasets, the expression data were compared to the MDA-MB-231 RNA-seq data from the Cancer Cell Line Encyclopedia, which showed a correlative expression signature [36].

### 2.13. Statistical Analysis

Statistical analyses were performed using the Mann–Whitney U test for comparing population medians of migrating cells for unpaired samples while Wilcoxon signed-rank test were used for paired samples. For comparison of tensile stiffness of different cell types, an ANOVA test followed by Tukey’s HSD test was used. A two-tailed t-test was used to compare the lamellipodia actin signal after EGF treatment. Effect size calculations of treatments were done by a logistic regression analysis. All analyses were performed in R software (Version 4.0.2, R Foundation for Statistical Computing, Vienna, Austria).

## 3. Results

### 3.1. MB-231 Cells and Their Bone-Seeking Derivatives Exhibit a Broad Spectrum of Single Cell Morphotypes

**Figure 1:**
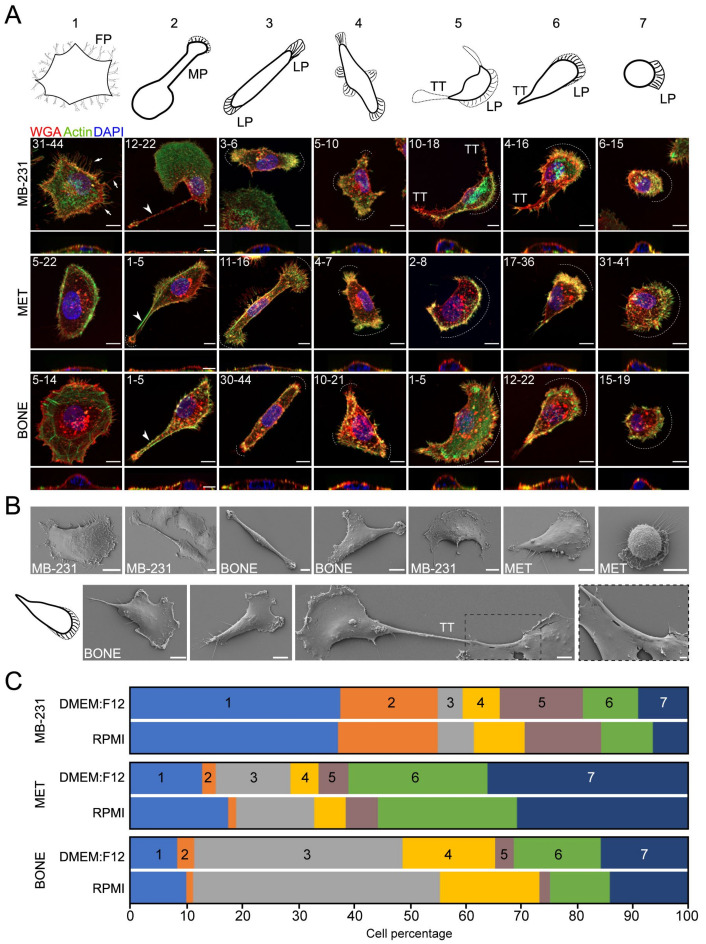
Breast cancer cells exhibit seven distinct morphologies. (**A**–**C**) The parental cells MB-231 and its bone-seeking derivatives, MET and BONE, were cultured on fibronectin-coated glass coverslips in DMEM:F12-based (**A**–**C**) or RPMI-based (**C**) media and processed either by cytochemistry (**A**) or SEM (**B**). Cells were cell-surface labeled with fluorochrome-conjugated WGA, and after cell permeabilization, stained with fluorochrome-conjugated phalloidin to highlight the actin filaments. Nuclei were counterstained with DAPI. Labeled samples were observed by structured illumination microscopy. Composites of 15–35 optical x–y sections (top panels) or a single x–z section (bottom panels) are displayed (**A**). Arrows indicate filopodia (FP), while the arrowhead the magnupodium (MP). Dotted lines show lamellipodia (LP). Note that the trailing-tail (TT) can be narrow, wide, or in contact with adjacent cell ((**B**), bottom panels, see inset highlighted with a dash box). The different morphotypes observed by fluorescence and illustrated in Models 1–7 were quantified (**A**,**C**). At least 100 cells per experiment were quantified (*n* = 3) and the maximum variation as indicated in percentage (**A**) or the average (**C**) of the cells with a given morphotype are presented for each cell line. Scale bars, 10 μm; 2 μm ((**B**), inset).

To study the morphological difference between parental triple-negative MB-231 and bone-colonizing variants MET and BONE at the single cell level, cells were cultured on fibronectin-coated substrates in a subconfluent state, and then labeled with fluorochrome-conjugated WGA to stain the cell surface and phalloidin to stain the actin filaments (Figure 1A). Morphological findings were validated by SEM (Figure 1B, top panels). Among the three cell lines, seven distinct cell morphologies were observed. In Morphotype 1, cells were large and spread, often with short finger-like filopodia at their periphery. They did not show any form of polarization. In Morphotype 2, cells harbored a thick and long protrusion, referred to as a magnupodium, which extended several micrometers from the cell body. Often, a sheet-like lamellipodium emerged from its terminal point, while part of its structure was not fully in direct contact with the substrate. In Morphotypes 3 and 4, cells had an elongated fibroblastic-like phenotype (i.e., spindle-shaped morphology) with the presence of small lamellipodia either at each end (#3) or on the side of their lateral borders (#4). In Morphotypes 5 and 6, cells contained a large, actin-rich lamellipodium where “trailing tail-like” structures were detected either at cell periphery (#5) or at the rear pole, i.e., at 90 degrees to the lamellipodium (#6), respectively. In the latter case, two to three smaller lamellipodia might be observed, instead of one large one. The trailing tail can also be narrow, wide, and, in some cases, in close contact with an adjacent cell (see examples with BONE cells; Figure 1B, bottom panels). Morphotypes 5 and 6 were, respectively, reminiscent of migrating keratocytes and fibroblasts [37]. In Morphotype 7, the cells were small with a round shape and a large lamellipodium was present. They often contained short filopodia (Figure 1A,B).

The abundance of these morphotypes varied considerably among the cell lines. While highly abundant in MB-231 cells (50% of population), Morphotypes 1 and 2 were observed less frequently in bone-seeking variants (Figure 1C). On contrast, those with an elongated morphology (i.e., Morphotype 3) were highly enriched in BONE cells. A gradually ascending frequency of Morphotype 3 from MB-231 to MET to BONE cells was observed. Cells with a migratory-like phenotype (i.e., Morphotypes 5–7) were prevalent either in MB-231 (#5) or in MET and BONE (#6) populations. Morphotype 7 was more frequently observed in bone-seeking cells. The individual morpho-profiles of all cell lines were stably reproducible among a different culture medium and among different cell passages (Figure 1C, data not shown).

Altogether, these experiments revealed a stable spectrum of common and differential morphotypes among MB-231 cells and their bone-seeking derivatives. While cells with migratory phenotypes were prevalent among all cell lines, non-polarized large cells and those with magnupodium were enriched in parental MB-231, whereas cells with an elongated morphology tended to accumulate in the enhanced osteotropic cell lines MET and BONE.

### 3.2. Differential Motility of Breast Cancer Cells with Specific Morphotypes

**Figure 2:**
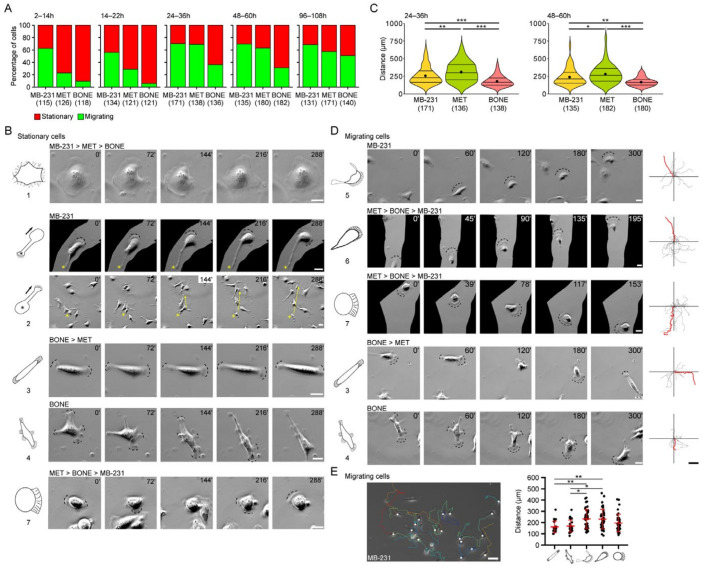
The parental breast cancer cells MB-231 and its bone-seeking derivatives, MET and BONE, exhibit different motility behaviors. (**A**–**E**) Cells were cultured on fibronectin-coated glass bottom dishes for a given period of time (2, 14, 24, 48 and 96 h) before being analyzed by time-lapse video microscopy for a continuous period of 12 h. The percentage of cells that are or not migrating during the entire analysis period is presented (**A**). Number of cells analyzed for each condition is indicated in parentheses. Examples of cells with a given morphotype without (**B**) or with (**C**–**E**) motility are presented. Cell lines enriched with a given morphotype are indicated above the phase-contrast micrographs, while the elapsed time of the movie is shown in the right corner. In some cases, a black area was drawn to focus on the cell of interest. In cells with Morphotype 2, the asterisk indicates the anchor point and the arrow shows the extension of the magnupodium (**B**). Dashed lines indicate lamellipodia. The distance travelled by migrating cells was determined and presented in the violin plots where the horizontal lines from bottom to top indicate the first, median and third quartile, respectively, while the mean for each population is represented by a dot (**C**). The motility of cells with a given morphotype is presented in the phase-contrast micrographs and their migration as either tracking diagrams ((**D**), 10 individual cells are displayed) or graphs ((**E**), *n* > 20 cells, mean and standard deviations are presented). An example of the cell tracking from a time-lapse video is presented (**E**). The red line in the tracking diagram indicates the cell displayed in the corresponding images. Note that motile cells with Morphotypes 5 and 6 display the higher motility (**D**,**E**). The images (**B**,**D**) presented are excerpts from the Videos S1 to S11. Means of each population were compared using Mann–Whitney U tests. * *p* < 0.05; ** *p* < 0.01; *** *p* < 0.001. Scale bars, 10 µm (micrograph, (**B**,**D**)); 100 µm (tracking diagram, (**D**)); 50 µm (**E**).

Cells were seeded on fibronectin-coated glass coverslips and cultured up to 108 h. At various time points, cell migration was recorded for a period of 12 h using time-lapse video microscopy. We defined a cell as migrating if, during recording, it displaced more than 60 μm, i.e., the distance corresponding to the length of about three cells. Cells that remained within this limit were considered stationary. MB-231 cells adhered very quickly on the substrate and started to migrate. Regardless of the time elapsed after seeding (i.e., 2, 14, 24, 48 and 96 h), approximately 60–75% of the cells were observed to be migratory (Figure 2A). This contrasts sharply with MET and BONE cells, of which less than 30% and 10%, respectively, moved within the first 24 h of incubation, suggesting a delay in cell-matrix adhesion. After this period, MET cells resembled MB-231 cells in their motility, while the proportion of migrating BONE cells remained lower throughout the entire observation period. At 96–108 h post-seeding, all cell lines were motile, while population density was reaching confluence, which showed the ability of migration within a monolayer of cells.

Subsequently, we compiled migration behavior of each cell line with the underlying single cell morpho-profile. We observed that stationary cells harbor different morphotypes (i.e., Morphotypes 1 to 4 and 7). The large cells with Morphotype 1 did not move per se. In those with Morphotype 2, the magnupodium emerging from the cell body could be generated either as the cell body itself shifts (similar to a trailing tail) or as an expanding protrusion (Figure 2B and Videos S1–S3). The lifetime of the former could be long (up to several hours) or short, as observed for the latter, in which the lamellipodium at its extremity favors its extension. Subsequently, the whole cell body can move towards it (see below). A fraction of the cells with Morphotypes 3, 4 and 7 showed no displacement, although they were highly dynamic due to the formation of multiple lamellipodia in various opposite directions, indicating a lack of coordination between the growth of the front end of the cell and the retraction of the back end (Figure 2B and Videos S4–S6).

By analyzing the individual motile cells, we found that the MET cell population is composed of the cells that traveled the greatest distance independently of the incubation period after seeding (e.g., between 24–36 h; 329 ± 14 μm, mean and standard error of the mean). On the contrary, BONE cells had a lower motility that appears in the whole population (181 ± 12 μm between 24–36 h), as shown in the violin plots (Figure 2C). MB-231 cells have an intermediate migrating distance under the same condition (262 ± 12 μm). These data were consistent with the distinct cell morphologies observed among the three cell lines. As displayed with tracking diagrams and graphs, cells with Morphotypes 5 and 6 were those for accounting of the greater motility and total migration distance, while those with Morphotypes 3 and 4 had lower motility (Figure 2D,E, see Videos S7–S11). However, the latter could adopt transient and reversible morphologies similar to those of Morphotypes 5 and 6, which might explain their displacement. It is interesting to note that cells with a given morphotype did not change their general morphology over time, suggesting that the single cell morphotypes were stable. It should be mentioned that a magnupodium can transiently emerge from MB-231 cells with Morphotypes 4–6, a phenomenon that was not observed with MET and BONE cells under these conditions (see below). Thus, cells of Morphotypes 5 and 6 adopt a mesenchymal mode of migration, whereas those of Morphotype 7 adopt an amoeboid mode, characterized by weak adhesions and increased cell contractility.

Taken together, we observed that MB-231 cells generally exhibit a heterogeneous spectrum of motility, which is markedly altered in the bone-seeking derivatives MET and BONE. Moreover, we show that single cell morphotypes differ in their motility and their modes of migration.

### 3.3. MET Cells, but Not MB-231 and BONE, Form Colonies That Affect Their Motility

**Figure 3:**
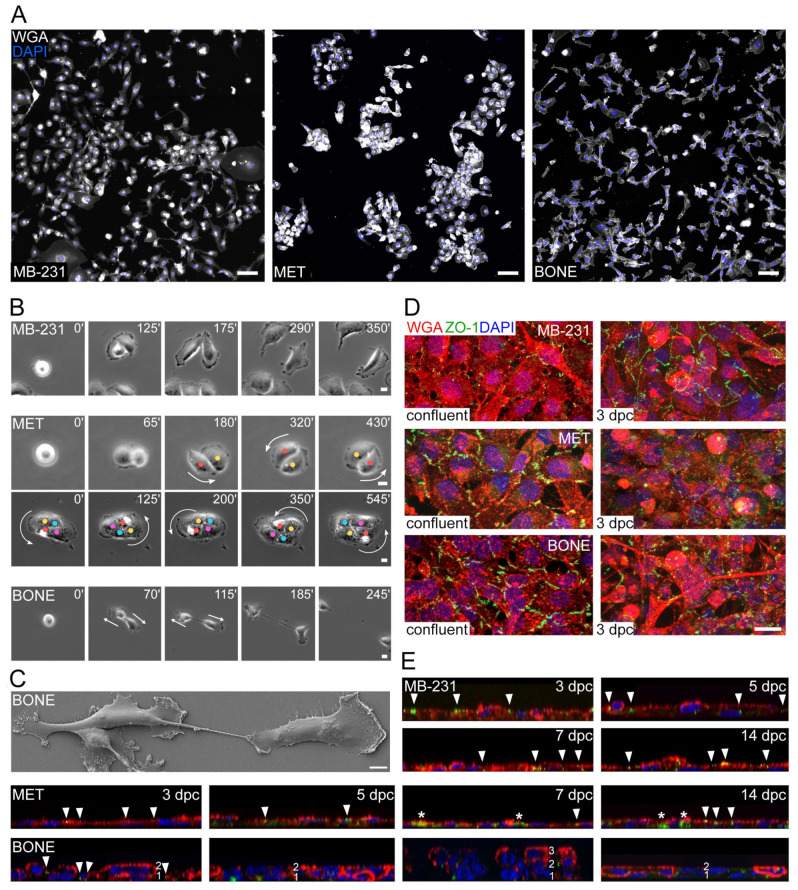
The parental breast cancer cells MB-231 and its bone-seeking derivatives, MET and BONE, display different features upon confluence. (**A**–**E**) Three cell lines, as indicated, were grown on fibronectin-coated glass coverslips from 70% confluence (**A**–**C**) to various states of confluence (**D**,**E**), as indicated by the day of post-confluence (dpc), before being analyzed by immunocytochemistry (**A**,**D**,**E**), time-lapse video microscopy (**B**) and SEM (**C**). Cells were cell-surface labeled with fluorochrome-conjugated WGA alone (**A**), or upon cell permeabilization, immunolabeled for ZO-1 (**D**,**E**). Nuclei were counterstained with DAPI. Cells were observed either by epifluorescence (**A**) or structured illumination microscopy (**D**,**E**). Composites of 15–45 optical x–y sections (**D**) or a single x–z section (**E**) are displayed. Arrowhead indicates the presence of ZO-1 at the cell junctions, while asterisk shows its intracellular localization (**E**). Numbers (1–3) indicate the multiple cell layers observed in the BONE population (**E**). The phase-contrast micrographs extracted from individual movies are presented (**B**). The elapsed time is shown in the right corner. The individual cells in the MET population were marked to follow their position at different time points. The arrow indicates the direction of cell movement. The images shown are displayed in Videos S12–S15. Scale bars, 100 µm (**A**); 10 µm (**B**,**C**); 20 µm (**D**).

As we previously had analyzed cells at the single cell level, we subsequently studied cellular morphology under confluent culture conditions. Interestingly, as cell confluence increased, MET cells began to form compact colony-like structures, whereas BONE cells grew in a more dispersed manner on the culture dish and exhibited a chain-like appearance, consistent with the predominant Morphotype 3 (Figure 3A). In contrast, MB-231 cells showed a mixture of phenotypes with individually growing cells or cell clusters that were often linked by magnupodia. Time-lapse video microscopy revealed that these phenotypes were closely related to the behavior of cells upon division, where daughter MET cells, unlike MB-231 and BONE cells, often remained attached to each other and rotated collectively (Figure 3B and Videos S12–S15). This contact-dependent migration may explain the high amount of migration within a confluent layer, in which cells can move longer distances without losing cell–cell contacts (see above). It remains to be determined whether this phenomenon can result in collective cell migration. After cell division, the midbody that links the two daughter BONE cells was often apparent in the last step of cytokinesis, in contrast to MB-231 and MET cells (Figure 3B,C).

The analyses of the cells at different confluent states revealed profound differences between the three cell lines. To evaluate the polarization of the cell monolayer, we double-labeled them with WGA and Zonula occludens-1 (ZO-1), which is located at the contact zone between cells [38]. At the first state of confluence, MET and BONE cells rapidly developed junctions between them, as illustrated with ZO-1 immunostaining (Figure 3D). In contrast, MB-231 cells showed a delay in this process, where most of the intercellular interactions occurred only 3 days after reaching the post-confluent state. The latter phenomenon remained constant in the MB-231 cell monolayer at all states of analysis (i.e., up to 14 days after confluence) (Figure 3E). Surprisingly, neither MET nor BONE cells maintained a tight cell–cell contact, and ZO-1 was redistributed from the cell membrane to the cytoplasmic compartment after 3 days post-confluence (Figure 3D,E). In addition, the BONE cells began to develop multiple cell layers, indicating an epithelial cell polarization defect (Figure 3E). The immunoblot of ZO-1 confirmed its expression in all cell lines and its reduction upon confluence (Figure S1A). We also probed for cadherin proteins that mediate calcium-dependent homotypic cell–cell adhesion. Immunoblots of E-cadherin (CD324) and N-cadherin (CD325) revealed a very weak expression of the former in MB-231 and MET cells, and the absence of the latter in all cell lines (Figure S1B). The CD324 expression also seems to depend on the confluent state of the cells (Figure S1B). The decreased expression of CD324 has been correlated with increased invasiveness of breast cancers [39,40,41], and the low and/or absence of CD324 and CD325 expression in MB-231 cells are in agreement with previous reports [42,43,44]. The complete absence of CD324 in BONE cells may explain the formation of multiple cell layers.

Collectively, we observed that bone-seeking cells do not only acquire changes in the level of morphology and motility at the single cell level, but also at the level of cellular interactions among confluent and post-confluent states. Moreover, bone-seeking clones were associated with a loss of epithelial features and loss of monolayer organization.

### 3.4. Differential Effects of CXCL12 and EGF on Cell Motility and Morphotype Distribution

**Figure 4:**
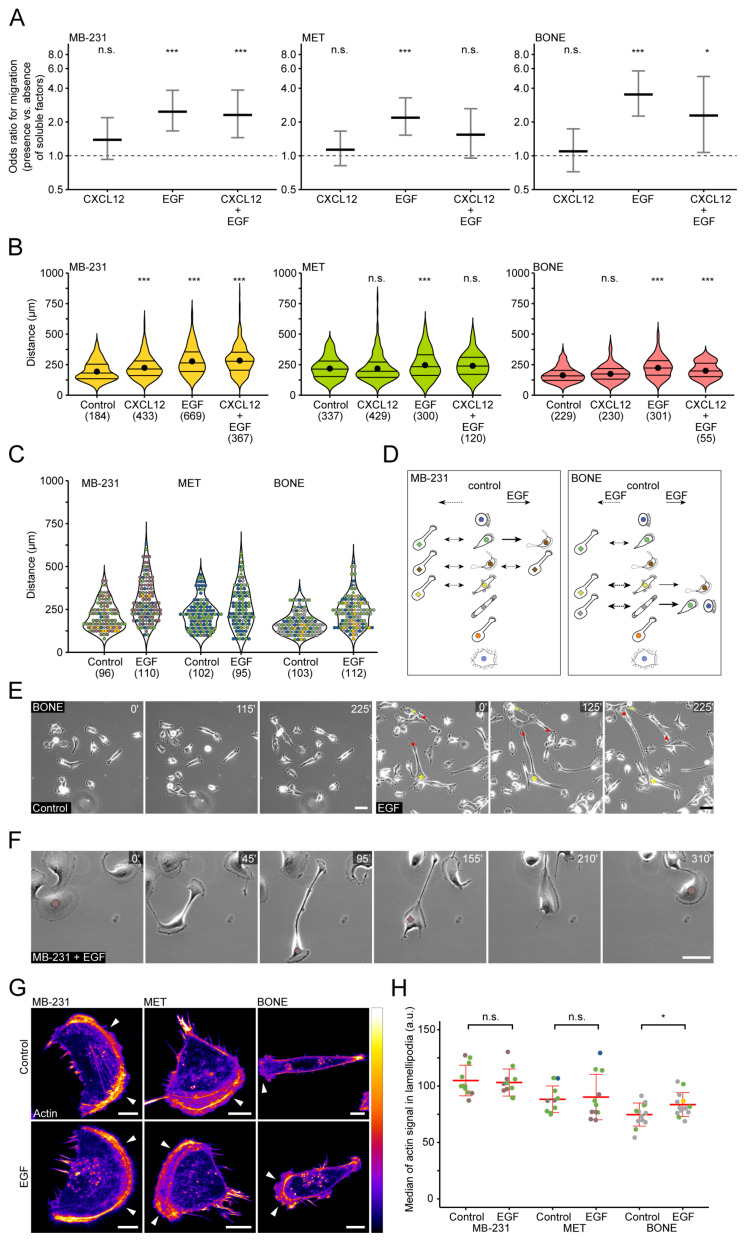
Impact of CXCL12 and EGF on the migration of breast cancer cells. (**A**–**F**) The parental breast cancer cells MB-231 and its bone-seeking derivatives, MET and BONE, were incubated in the absence (control) or presence of CXCL12 (100 ng/mL) or EGF (25 ng/mL) alone or in combination, and monitored by time-lapse video microscopy over a period of 12 h (i.e., 48–60 h after seeding, see Figure 2 legend). The odds ratios and their 95% confidence interval indicating the change in the migration behavior of cells compared to control (value 1, dashed line) are displayed in the graphs ((**A**), *n* = 3–4). The distance travelled was determined for each individual cell and presented in the violin plots (**B**). The horizontal lines from bottom to top indicate the first, median and third quartile, respectively, while the mean for each population is represented by a dot. Number of cells analyzed for each condition is indicated in parentheses. Medians of each population were compared against the control using Wilcoxon signed-rank tests. The distance travelled by about 100 individual cells in the absence or presence of EGF was further dissected according to their morphotypes (**C**). Color code and morphological changes are presented (**D**). Time-lapse microscopy showed the transient generation of a magnupodium by a BONE or MB-231 cell after the EGF treatment (**E**,**F**). Unlike MB-231 cells (**D**), untreated BONE cells (control) did not show this phenomenon (**E**). The asterisk and the red dot indicate respectively the stationary and the moving part of the cell (**E**). The elapsed time is shown in the right corner. (**G**,**H***)* Cells were incubated in the absence (control) or presence of EGF for 6 h, PFA-fixed and stained with fluorochrome-conjugated phalloidin. Fluorescent images revealed the concentration of actin filaments at the leading edge of breast cancer cells ((**G**), arrowhead). The fluorescence signal (strong to weak) is shown on the right sidebar. Quantification of F-actin at the front edge of lamellipodium of cells treated without or with EGF (**H**). The mean and standard deviation are presented, and each point represents an individual cell with a given morphotype as indicated in panel D (*n* = 10 cells). Data were analyzed using a two-tailed *t*-test. N.s., not significant; * *p* < 0.05; *** *p* < 0.001. Scale bars, 50 µm (**E**,**F**); 10 µm (**G**).

In the bone marrow, the chemokine CXCL12 (i.e., stromal-derived factor-1) recruits hematopoietic stem cells through its interaction with CXCR4 receptor (CD184), and this axis can be hijacked by cancer cells [10,45]. To determine the impact of soluble factors on breast cancer cell motility, we added either CXCL12 or epidermal growth factor (EGF), or a combination of both, to our cell cultures just prior to time-lapse video recording. EGF has previously been shown to influence MB-231 cell motility and its signaling has been correlated with the cellular progression towards invasion [46,47]. Surprisingly, the number of migrating cells did not significantly increase in all three cell lines upon addition of CXCL12 (100 ng/mL) compared to control cells, i.e., without chemokines (Figure 4A). However, the migration distance significantly increased in MB-231 but not in MET and BONE (Figure 4B). The latter observation was in line with the CXCR4 expression levels observed by immunoblots (Figure S1C). In the subconfluent and confluent states, CXCR4 was strongly expressed in the MB-231 cells, but virtually absent in others. Furthermore, the number of CXCR4^+^ cells varied significantly between these cell line populations. In contrast, the addition of EGF (25 ng/mL) increased the migration of cells as well as the migratory distance in all populations (Figure 4A,B). Consistent with these findings, the EGF receptor was strongly detected in all three cell lines, with highest expression in BONE (Figure S1D). No additive effect, barring a slight counter-negative effect in MET and BONE, was observed after combined stimulation with CXCL12 and EGF (Figure 4A,B).

Time-lapse video recording combined with morphotype analysis revealed that the cells with the Morphotype 6 in the BONE cell population exhibited the greatest motility when cultured in the presence of EGF (Figure 4C). Their number was also increased while those with Morphotype 3, and to a lesser extent 4, were reduced, suggesting that EGF modifies the morphotype distribution and motility of BONE cells (Figure 4C,D). The morphotype shifting was not evident in MB-231 and MET cells with the exception of Morphotype 6 in MB-231 cells, which is reduced concomitantly with an increase of Morphotype 5 (Figure 4C,D; data not shown). EGF treatment also favored the transient formation of a magnupodium from BONE cells with Morphotypes 3, 4 and 6 (Figure 4D,E). As mentioned above, spontaneous appearance of magnupodium occurred in MB-231 cells but not in MET cells in the absence or presence of EGF (Figure 4D,F; data not shown). It is known that EGF pathways can activate actin-related protein (Arp) 2/3 complex-dependent actin polymerization [48,49], which is important for the generation of membrane extensions such as lamellipodia. Interestingly, the amount of actin at the leading edge of EGF-treated motile BONE cells was significantly increased, consistent with their enhanced cell migration (Figure 4G,H). ARPC2 protein, a component of the Arp2/3 complex, was similarly expressed in all three cell lines (Figure S1D).

Taken together, we demonstrate that the migratory response to CXCL12 was only observed in parental MB-231 but not in any of the bone-seeking cells. Moreover, all investigated cell lines readily responded to EGF with migration. Particularly in BONE cells, EGF additionally induced a shift in the morphotype profile, represented by the formation of magnupodia and directed lamellipodia.

### 3.5. Differential Expression of Selective Cluster of Differentiation Molecules among Breast Cancer Cell Sub-Lines

**Figure 5:**
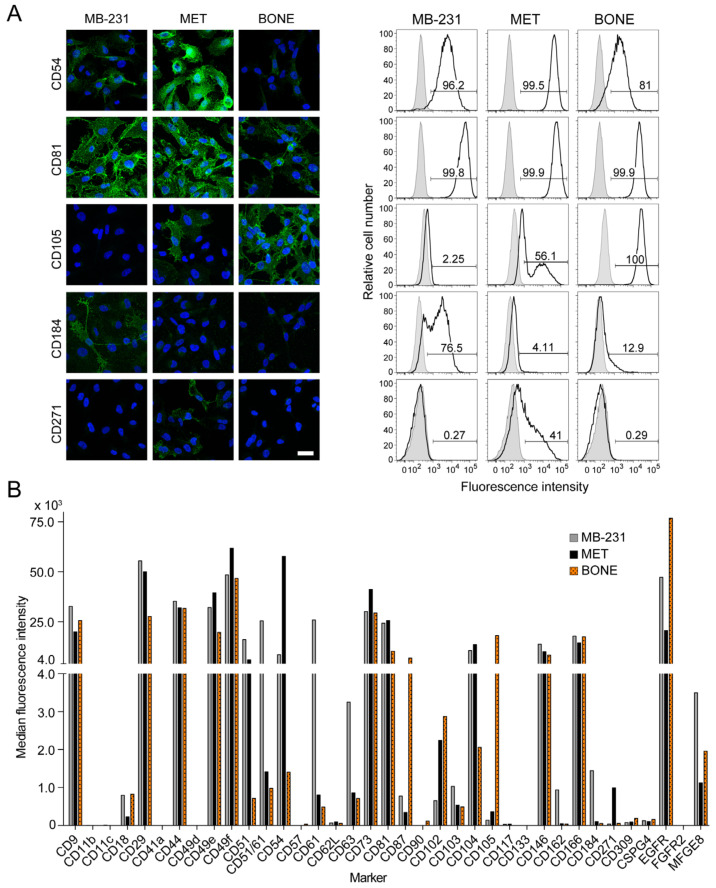
Differential expression of CD markers between breast cancer cell lines. (**A,B**) The parental cells MB-231 and its bone-seeking derivatives, MET and BONE, were cell-surface immunolabeled for one of a panel of CD markers and other proteins as indicated prior to cytochemistry ((**A**), left panels) and flow cytometry ((**A**), right panels, (**B**)) analyses. See also Figure S2. Percentages of positive cells are indicated in the histograms (**A**) and the median fluorescence intensity is presented for each marker (**B**). Representative immunocytochemistry and flow cytometry experiments acquired under uniform instrument setting for all cell lines are displayed. Scale bar, 25 μm.

Intercellular adhesion proteins and integrins play a central role in cell-to-cell and cell-to-extracellular matrix (ECM) adhesions, and thus impact the actin organization axis that regulates cancer cell progression and metastasis [50]. To further understand the abovementioned differential features of bone-seeking cells with regard to their migration and adhesion, we profiled cell surface expression of various CD markers by immunofluorescence and/or flow cytometry (Figure 5A,B and Figure S2).

The intercellular adhesion molecule 1 (ICAM1/CD54) was reduced in BONE cells compared to MB-231 and MET cells, while the intercellular adhesion molecule 2 (ICAM-2/CD102) was strongly expressed in both bone-seeking sub-lines by comparison to MB-231 cells (Figure 5A,B). Among the integrins, ITGAM (CD11b), ITGAX (CD11c), ITGA2B (CD41a) and ITGA4 (CD49d) were negative in all cell lines while ITGB2 (CD18), ITGB1 (CD29), ITGA5 (CD49e), ITGA6 (CD49f), ITGAV (CD51), ITGAV/ITGB3 (CD51/CD61), ITGB3 (CD61), ITGAE (CD103) and ITGB4 (CD104) were positive (Figure 5B and Figure S2). Interestingly, expression levels of ITGA5 and ITGB1, i.e., the components of fibronectin receptor (α5β1 heterodimer), in MET and BONE cells were different compared to parental cells MB-231 (Figure 5B, see also Figure S1E). This could explain, at least in part, the delay in cell–matrix adhesion, and ultimately their differential migration.

Of the tetraspanin proteins, CD9 expression was slightly higher in MB-231 cells, whereas CD81 was reduced in BONE cells compared to MB-231 and MET cells (Figure 5A,B, see also Figure S1F). CD9, also known as Motility-Related Protein-1, and CD81 can modulate cell adhesion and/or migration. Lysosome-associated membrane protein 3 (LAMP3/CD63) was markedly decreased in both MET and BONE cells (Figure 5B). Across other CD markers, P-selectin glycoprotein ligand-1 (PSGL-1/CD162) was decreased in both MET and BONE cells (Figure 5B), while the urokinase receptor (uPAR/CD87) and endoglin (CD105) were notably increased in BONE cells (Figure 5A,B). Three subpopulations of CD105—negative, low and high—were found in the MET cell line, which is in sharp contrast to MB-231 and BONE cells, where the former and latter are predominantly negative or positive, respectively (Figure 5A,B). The low-affinity nerve growth factor receptor (LNGFR/p75NTR/CD271), which could play a role in cell migration as a regulator of actin assembly [51], was selectively elevated in MET cells. Milk fat globule-EGF factor 8 protein (MFGE8/lactadherin) was higher in MB-231 cells (Figure 5B). The secreted MFGE8 is the ligand for αVβ3-5 integrins and has been shown to influence the viability, invasion and migration of breast cancer cells [52,53]. The expression of CD44, 5′-nucleotidase (NT5E/CD73), melanoma cell adhesion molecule (MCAM/CD146) and ALCAM (CD166) were similarly expressed in all cell lines, while 3-beta-glucuronosyltransferase (B3GAT1/CD57), L-selectin (CD62L), Thy1 (CD90), KIT (CD117), prominin-1 (CD133), vascular endothelial growth factor receptor 2 (VEGFR-2/CD309), chondroitin sulfate proteoglycan 4 (CSPG4) and FGFR2 were low or absent (Figure 5B and Figure S1F).

Interestingly, we observed heterogeneity in CXCR4 expression in MB-231 cells, which represent subpopulations with a negative, low and high expression, whereas only a minute fraction of bone-seeking cells weakly express CXCR4 (Figure 5A,B), in agreement with the immunoblot (Figure S1C). This could explain the lack of functional response of the latter to the addition of CXCL12. EGF receptor expression was stronger at the BONE cell surface, which is in agreement with immunoblot data (Figure S1D), and their migratory response to EGF (Figure 4A–C).

In addition to cell surface proteins, we analyzed the expression of cytoplasmic proteins. Using a pan-cytokeratin antibody, we observed a general reduction of it in BONE cells, whereas the alpha smooth muscle actin (SMA/ACTA2) was slightly increased in both MET and BONE cells (Figure S3A). Nestin was expressed at different levels among cells in the parental line MB-231, suggesting the presence of a distinct subpopulation, while being reduced in bone-seeking sub-lines (Figure S3A). Interestingly, vimentin was highly expressed in all cell lines and appeared to be concentrated at the magnupodium, whereas SMA was expressed at their tips (Figure S3A).

In summary, we highlight marked differences of bone-seeking cells compared to MB-231 with regard to the expression profile of integrins and other relevant adhesion molecules, which could explain their differential morphology and motility.

### 3.6. Differential Surface Glycosylation Patterns among Breast Cancer Cell Sub-Lines

**Figure 6:**
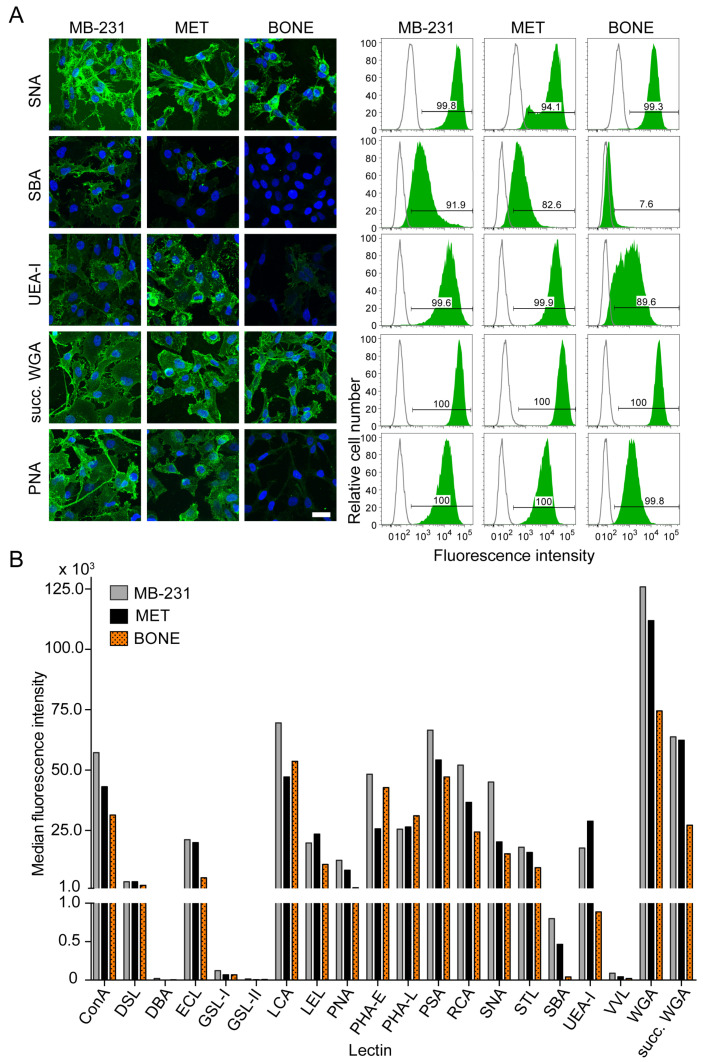
Differential binding of lectins to breast cancer cell lines. (**A**,**B**) The parental cells MB-231 and its bone-seeking derivatives, MET and BONE, were cell-surface labeled with one of a panel of distinct FITC-conjugated lectins prior to cytochemistry ((**A**), left panels) and flow cytometry ((**A**), right panels, (**B**)) analyses. See also Figure S4. Percentages of positive cells are indicated in the histograms (**A**) and the median fluorescence intensity is presented for each lectin (**B**). Data from representative experiments acquired under uniform instruments setting for all cell lines are displayed. The lectins used were Concanavalin A (ConA), Datura Stramonium Lectin (DSL), Dolichos Biflorus Agglutinin (DBA), Erythrina Cristagalli Lectin (ECL), Griffonia Simplicifolia Lectin I (GSL-I), Griffonia Simplicifolia Lectin II (GSL-II), Lens Culinaris Agglutinin (LCA), Lycopersicon Esculentum Lectin (LEL), Peanut Agglutinin (PNA), Phaseolus Vulgaris Lectin E (PHA-E), Phaseolus Vulgaris Lectin L (PHA-L), Pisum Sativum Agglutinin (PSA), Ricinus Communis Agglutinin I (RCA), Sambucus Nigra Lectin (SNA), Solanum Tuberosum Lectin (STL), Soybean Agglutinin (SBA), Ulex Europaeus Agglutinin I (UEA-I), Vicia Villosa Lectin (VVL) and Wheat Germ Agglutinin (WGA, succinylated (succ)-WGA). Scale bar, 25 μm.

The glycosylation moieties of proteins can significantly alter their cellular functions [54]. Therefore, we investigated the general cell surface glycan pattern of the three cell lines by probing them with several FITC-labeled lectins (for abbreviations, see Figure 6 legend). The labeled cells were analyzed by cytochemistry and flow cytometry (Figure 6 and Figure S4). ConA, DSL, ECL, LCA, LEL, PNA, PHA-E, PHA-L, PSA, RCA, SNA, STL, UEA-I, WGA and succ-WGA were positive in all cell lines. The intensity of lectin binding varied slightly between cell lines. A significant difference was observed for SBA, UEA-I and PNA, where BONE cells showed a weaker or no signal (Figure 6A,B). VVL showed binding to a few cells (about 20%) in the MB-231 population, but almost none in MET and BONE cells. In contrast, DBA, GSL-I and GSL-II were negative in all cell lines (Figure 6B).

Conclusively, a differential pattern of surface glycosylation is observed in bone-seeking cells, pointing to an additional layer of complexity within an osteotropic phenotype of breast cancer cells.

### 3.7. Phenotypic Comparison of Bone vs. Brain Metastatic Breast Cancer Cells

**Figure 7:**
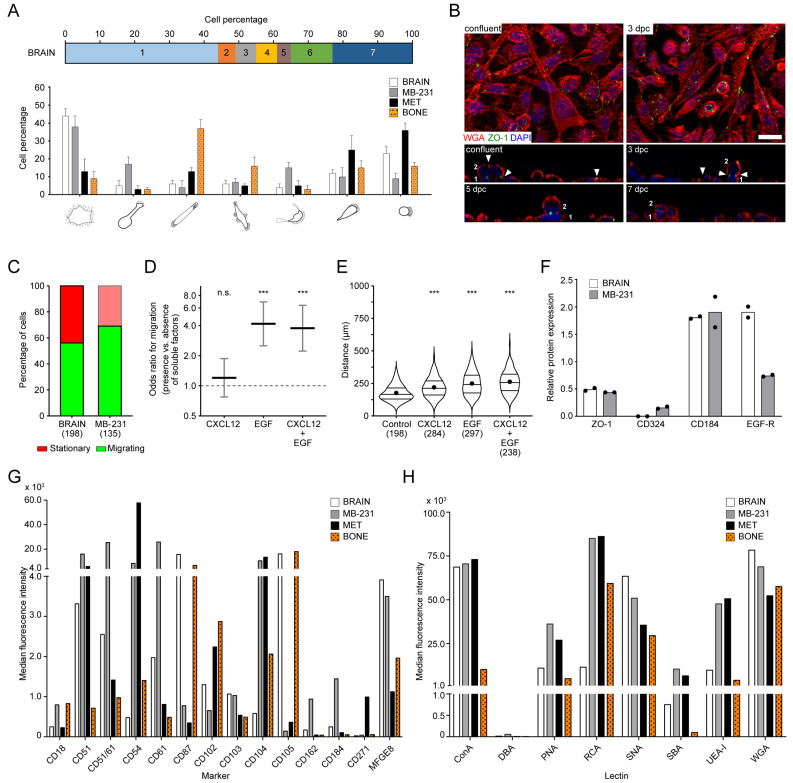
Characteristics of breast cancer, brain-seeking metastatic variant. (**A**–**E**) BRAIN cells were cultured on fibronectin-coated glass coverslips under subconfluent (**A**,**C**–**E**) or post-confluent (**B**) states, as indicated by the day of post-confluence (dpc), and processed by cytochemistry (**A**,**B**) or time-lapse video microscopy for a continuous period of 12 h after 48 h of plating (**C**–**E**). For the cytochemistry, they were cell-surface labeled with fluorochrome-conjugated WGA, and after cell permeabilization, stained with fluorochrome-conjugated phalloidin to highlight the actin filaments ((**A**), data not shown) or immunolabeled for ZO-1 (**B**). Nuclei were counterstained with DAPI. The samples were observed by structured illumination microscopy and presented as described in the legends of Figure 1 and Figure 3. The quantification of cells with a given morphotype (1–7) is presented ((**A**), upper panel) and the mean and standard deviation are given (lower panel, >100 cells per experiment were analyzed, *n* = 3). For comparison, the data obtained with the parental (MB-231) and bone-seeking variants (MET and BONE) are displayed. The numbers (1 and 2) indicate the multiple cell layers observed in the confluent BRAIN cell population, while the arrowhead points to the presence of ZO-1 at the cell borders (**B**). For the time-lapse video microscopy, cells were incubated in the absence ((**C**–**E**), control) or presence of CXCL12 (100 ng/mL) or EGF (25 ng/mL) alone or in combination (**D**,**E**). The number of cells analyzed for each condition is indicated in parentheses. The percentage of cells that are or not migrating during the entire analysis period is presented (**C**). The odds ratios and their 95% confidence intervals, indicating the change in the migration behavior of cells compared to control (value 1, dashed line), are displayed in the graphs ((**D**), *n* = 3–4). The distance travelled was determined for each individual cell and presented in the violin plots (**E**). The horizontal lines from bottom to top indicate the first, median and third quartile, respectively, while the mean for each population is represented by a dot. Medians of each population were compared against control using Wilcoxon signed-rank tests. N.s., not significant; *** *p* < 0.001. (**F**,**G**) The expression of particular proteins in subconfluent BRAIN cells were analyzed by immunoblotting (**F**) or flow cytometry (**G**). See also Figure S2. The relative expression of a given protein was normalized to α-tubulin (**F**). The mean of 2 independent experiments is presented and each point represents the value of the individual experiment (**F**). The median fluorescence intensity is presented for each marker ((**G**), see Figure S2, data shown). Data from MB-231 cells or MET/BONE cells were used for comparison. (**H**) The binding of lectins. BRAIN cells (and others) were cell-surface labeled with one of a panel of distinct biotin-conjugated lectins followed by fluorochrome-conjugated streptavidin prior to flow cytometry analyses. The median fluorescence intensity is presented for each lectin. Data from representative flow cytometry experiments acquired under uniform instrument setting for all cell lines is displayed. Scale bar, 20 µm.

Brain-seeking cells MDA-MB-231-BrM2 cells (herein referred to as “BRAIN”) were analyzed with regard to their morphotypes and motility using the same procedures as described above. The overall distribution of Morphotypes 1 to 7 showed a similarity with MB-231 cells, with three exceptions (Figure 7A). Both BRAIN and MB-231 cells contained a larger proportion (about 40%) of cells with Morphotype 1. Cells with Morphotypes 3, 4 and 6 were present at the same proportion as MB-231 cells. In contrast to MB-231 cells, Morphotypes 2 and 5 were rare in BRAIN, while those with Morphotype 7 were more abundant. The latter characteristics were similar to bone-seeking cells. Cells with Morphotype 3 were more prevalent in MET and BONE (Figure 7A). Under confluent conditions, BRAIN did not form a monolayer, in contrast to MB-231 cells, and grew in multilayers like BONE (Figure 7B). The time-lapse video microscopy revealed that the proportion of migrating cells after 48 h of seeding was slightly lower than those observed in MB-231 cells, but the migrating ones showed a similar travelled distance (Figure 7C).

The addition of CXCL12 did not increase the proportion of migrating BRAIN cells, while EGF alone or in combination with CXCL12 did (Figure 7D). Nonetheless, the chemokine addition significantly increased the traveled distance, as in MB-231 cells (Figure 7E). The latter phenomenon was not observed with bone-seeking variants. No changes in cell morphology or transient magnupodium formation were observed in BRAIN after the addition of EGF (data not shown).

The expression of ZO-1, E-cadherin, CXCR4 and EGF receptor was analyzed by immunoblotting, while the antigenic profile of cell surface molecules was investigated by flow cytometry (Figure 7F,G and Figure S2). The expression levels of the total ZO-1 and CXCR4 were similar to parental MB-231 cells, while E-cadherin and EGF receptor mimicked BONE (Figure 7F). Among the integrins, flow cytometry revealed that ITGB1, ITGA5, ITGA6, ITGAV and ITGAV/ITGB3 were nearly all positive throughout the cell population (Figure 7G and Figure S2). The strong expression of both ITGAV and ITGB3 in BRAIN cells, just like in parental cells MB-231, contrasted with that of the bone-seeking sub-lines, notably in BONE cells in the case of ITGAV, similarly for ITGAE. At the opposite, ITGB4 was significantly reduced in BRAIN cells compared to all other cell lines. The ITGB2 showed a weak expression similar to MET cells. ITGAM, ITGAX, ITGA2B and ITGA4 were negative like in the other cell lines. Differential expression of ICAM1, uPAR, ICAM2, endoglin, PSGL-1, CXCR4, LNGFR and MFGE8 was also detected (Figure 7G). Notably, cell surface expression of ICAM1 and ICAM2 was less in BRAIN cells compared to the bone-seeking variants, while PSGL-1 was stronger. Similarly, CXCR4 at the cell surface was increased in BRAIN compared to MET and BONE, although it remained lower than in MB-231 cells (Figure 7G). Like MB-231 cells, MFGE8 was highly expressed in BRAIN.

Probing the BRAIN cells with lectins also revealed differences (Figure 7H). For instance, SNA, a lectin that binds to α2,6-linked sialyl groups, showed a strong staining in BRAIN compared to the bone-seeking sub-lines, which is in line with a previous study [21]. In contrast, the binding of RCA, a galactose-binding lectin, was decreased compared to all other lines. It was reported that RCA binds triple-negative breast cancer cells to a degree that is proportional to their metastatic capacities [55].

Altogether, some features appear to be common to both brain- and bone-seeking cells, but the overall cell morphology, migratory traits and molecular architecture characteristics are more similar between BRAIN cells and parental MB-231 cells, highlighting some biochemical aspects of breast cancer cells with increased osteotropic capacity.

### 3.8. Differential Biophysical Properties of Single Cell Morphotypes

**Figure 8:**
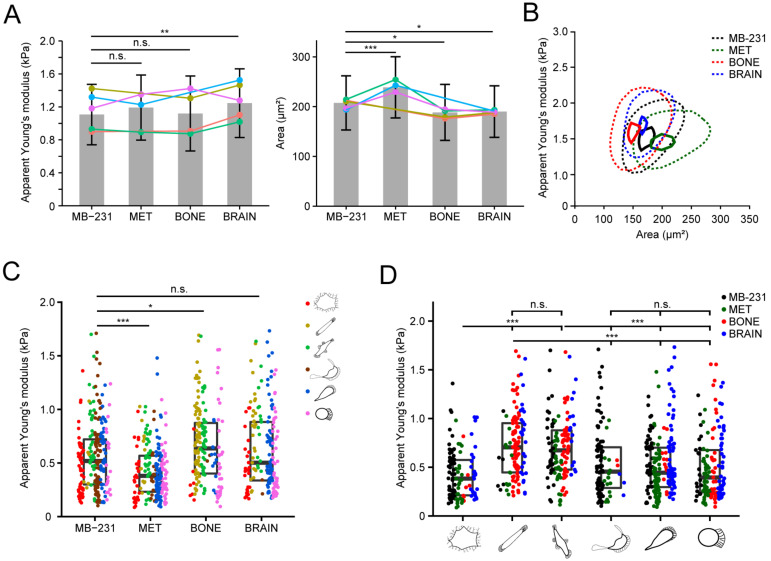
Biomechanical characteristics of breast cancer cells. (**A**) Real-time deformability cytometry analysis of MB-231 and its bone-colonizing (MET and BONE) and brain-colonizing (BRAIN) variants. The apparent Young’s modulus (left) and cross-sectional area (right) of the cells were calculated. Cell lines were measured sequentially for each individual experiment, indicated by the color of the points (*n* = 3–5 experiments). (**B**) The stiffness and area distribution for each cell line from a single representative experiment (*n* > 6300 cells per cell line). Solid inner line indicates 95% of the density while a dashed line indicates 50% of the density distribution. (**C,D**) Stiffness of adherent cells measured by atomic force microscopy. The overall stiffness for each cell line (**C**) or morphotype (**D**), independently of the morphotype or cell lines, respectively, were plotted with colors indicating the morphotypes or cell lines (**C**,**D**). Error bars indicate the standard deviation (**A**), and the center bar of the boxplots indicate the median while edges indicate the 25th and 75th percentiles, respectively (**C**,**D**). Population means were compared using Tukey’s test. n.s., not significant; * *p* < 0.05; ** *p* <0.005; *** *p* < 0.001.

Cell stiffness can play an important role in tumorigenicity and stemness of cancer cells, including their ability to metastasize [56,57]. This prompted us to determine the mechanical properties of the MB-231 cell line and its bone- and brain-seeking variants under suspensive and adherent conditions. To measure the cellular deformability in suspension, we performed RT-DC [28]. Calculation of the apparent Young’s moduli from cell deformability showed a modest increase in stiffness in BRAIN cells compared to MB-231 cells, which was persistent among replicates (Figure 8A,B). No difference in apparent Young’s moduli was observed in MET and BONE cells compared to MB-231 cells. Comparison of cell size in suspension revealed other differences: MET cells were larger, while BONE and BRAIN cells were slightly smaller than MB-231 cells (Figure 8A,B).

While RT-DC probes cells in suspension, adherent cells may exhibit further mechanical traits, which are guided by their interactions with the ECM and by the organization of the cytoskeleton and the F-actin cortex. To measure their mechanical properties during their adherent state, cells were locally indented by an AFM cantilever equipped with a spherical indenter. The obtained apparent Young’s moduli showed mechanical differences between the MB-231 derivatives, which were not observed by RT-DC measurement. MET cells were observed to be significantly more compliant than MB-231 cells, while BONE cells were stiffer (Figure 8C). The parental MB-231 cells and the brain-seeking variant were similar overall.

Classification of the biophysical cell traits under consideration of the single cell morphology revealed a significant variation in apparent Young’s moduli between morphotypes, particularly for the fibroblastoid Morphotypes 3 and 4, which were stiffer compared to the non-migrating cells of Morphotype 1 and highly migrating cells of Morphotypes 5, 6 and 7 (Figure 8D). The apparent Young’s moduli of a given morphotype were similar across all cell lines, suggesting that cell morphological traits are the major contributor to the overall stiffness profile of a given cell line.

Collectively, we show that single cell morphologies are associated with differential biophysical traits, particularly the cellular stiffness.

### 3.9. Transcriptional Profiling of Breast Cancer Cell Sub-Lines

**Figure 9:**
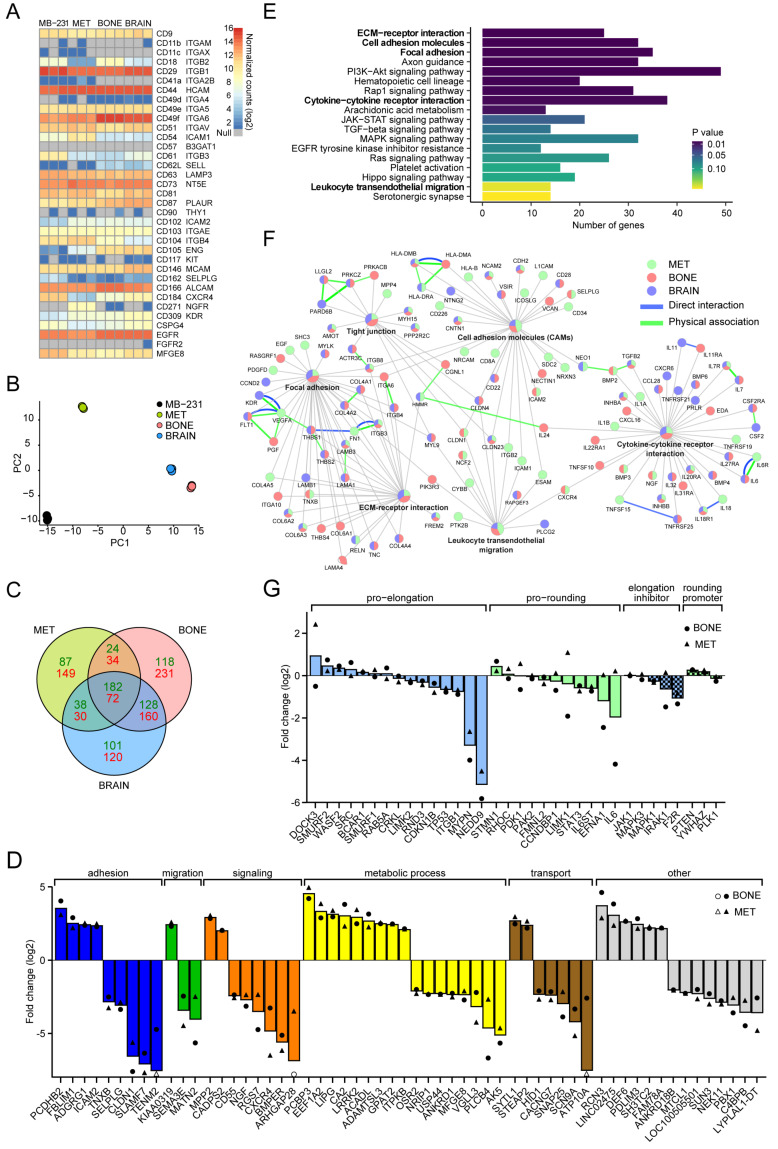
Transcriptomes of breast cancer cell lines—a comparison analysis. (**A**) Heatmap of selected genes where their protein products have been analyzed by immunofluorescence or flow cytometry (Figure 5 and Figure S2) is depicted. (**B**) Principal component analysis of the gene expression data of MB-231 and its bone-seeking (MET, BONE) and brain-seeking (BRAIN) sub-lines. (**C**) Global gene expression of MB-231 cells and its bone-colonizing (MET and BONE) and brain-colonizing (BRAIN) variants was analyzed by RNA-Seq. Venn diagram of the significantly differentially expressed genes in variants (MET, green; BONE, red; BRAIN, blue) compared to parental MB-231 cells is presented. Green and red numbers indicate upregulated and downregulated genes, respectively (log2 fold change > 2 or <−2). (**D**) Genes (58) which are significantly differentially expressed in both MET and BONE, but not in BRAIN compared to parental MB-231 cells are displayed. Bars represent the average of bone seeking sub-lines (MET and BONE) relative to MB-231 cells. Black triangle indicates expression in MET and black circle in BONE. Values which are outside of displayed range are indicated with empty symbols accordingly (TENM2 MET, 10.4; ATP10A MET, 12.5; ARHGAP28-BONE, 10.3). Color-coded bars represent genes associated with specific biological processes obtained from gene ontology lists: cell adhesion (GO:0007155), migration (GO:0016477), signaling (GO:0023052), metabolic process (GO:0008152) and transport (GO:0006810). The corresponding heatmaps are presented in Figure S5A. (**E**,**F**) Over-representation analysis of differentially expressed genes among cell lines (panel (**C**)) according to the KEGG pathways ((**E**), see Materials and Methods), and their network of interactions of the highlighted pathways is presented (**F**). The color code indicates in which cell line a given gene is differentially expressed compared to MB-231 cells. Blue lines indicate a direct interaction between gene products (e.g., ligand–receptor interaction), while green lines indicate those with a physical/structural association (e.g., within a protein complex). (**G**) Gene expression changes for selected genes in bone-seeking variants that impact the cell shape. The corresponding heatmaps are presented in Figure S5B.

By performing RNA-seq, we inquired whether the changes in bone-seeking cells at the level of single cell morphology, migration and molecular architecture are reflected by their transcriptomes. A strong correlation between the expression of a given transcript and its protein product was observed (Figure 9A and Figure S3B versus Figure 5 and Figure 7 and Figures S1, S2 and S3A). Differential protein expression between MB-231 cells and its sub-lines were confirmed at the mRNA level, indicating the robustness of our RNA-seq analysis. Although the migratory and phenotypic properties were somewhat similar between the two bone-seeking sub-lines MET and BONE, compared to the parental and brain-seeking cells, principal component analysis revealed BONE and BRAIN to have the highest similarity in their transcriptome (Figure 9B).

In order to define the gene expression signatures specific for bone-seeking cells, differentially expressed genes were retrieved using a stringent criterion (absolute log2-fold change > 2, *q* < 0.01) (Figure 9C). Compared to parental MB-231, 58 genes were differentially expressed in both MET and BONE (24 upregulated and 34 downregulated) but not in BRAIN (Figure 9C,D and Figure S5A). Most of these genes are involved in cellular adhesion (e.g., PCDHB2, ICAM2), migration and cell signaling (e.g., CADPS2, CXCR4) as well as in metabolism (e.g., LIPG, PLCB4). The former groups might favor the navigation to metastatic tissues, while the latter could favor colonization in the bone (Figure 9D). Similarly, 221 genes were differentially expressed in BRAIN but not in bone-seeking variants (Figure 9C and Figure S6).

Pathway analysis suggested that bone- and brain-seeking clones both dysregulate a variety of cellular pathways, particularly those associated with an ECM–receptor interaction, cellular/focal adhesion, cytokine receptor interaction, leukocyte transendothelial migration and tight junction pathways (Figure 9E). A list of genes categorized in these pathways is presented (Figure S7A). Moreover, 11 (e.g., CHSY3, FUT8, GALNT14, ST6GAL1) and 13 (e.g., GPRC5A, MMP14, PARP8) genes that are either associated with the glycan biogenesis or uncategorized with respect to these pathways showed a significant difference between the cell lines (Figure S7B,C, see also Figure S8). The former might explain the differential bindings of lectins to them, especially SNA lectin.

Analyzing the gene expression with less stringent filter criteria, we revealed further differentially expressed genes compared to those among the four cell lines, such as the molecules involved in adhesion and cellular navigation, including integrins (e.g., ITGA6, AD, B1-4 and B8), cadherins (e.g., CDH10, 11, and 18), claudins (CLDN1, 2, 4, 7, and 23), chemokine receptors and their ligands (e.g., CCR1, CCR7, CXCR4, CXCR6, and CXCL16) (Figure S8A–E). Certain components of the ECM, notably in the collagen (e.g., COL4A1, 4A2, 4A5, 4A6, 6A1-3, and 18A1) and laminin (e.g., LAMA1, B1, and B3) families, are also differentially expressed (Figure S8F). By resolving these signaling networks at the cell-line individual level (Figure 9F), a close relationship between specific gene expression signatures and altered adhesive and migratory functions of bone- and brain-seeking breast cancer cells became evident, which is consistent with our observations on the phenotypic, molecular and biomechanical properties of these cells.

Finally, to explain the differential morpho-profiles of bone-seeking cell lines compared to MB-231, we selectively drew attention to a series of genes that are known to regulate cell shape and are classified as pro-elongation or pro-contractility groups [58,59]. Their expression levels are thought to determine the morphology and the migration mode (i.e., mesenchymal versus amoeboid). Our data revealed that most of them were expressed at a similar level between all cell lines (Figure 9G and Figure S5B). One of them stands out, the neural precursor cell-expressed, developmentally downregulated 9 (NEDD9). This protein is particularly interesting since numerous publications have demonstrated its impact on the mode of migration of breast cancer cells. The NEDD9-associated pathway favors the elongated, mesenchymal mode of migration while inhibiting the rounded, amoeboid-such as migration (see Discussion). In such a context, RhoA downstream effector kinase ROCKII transcript, but not ROCKI, was elevated in both bone-seeking cells and BRAIN cells compared to MB-231 (data not shown). To sum up, we confirmed that changes in bone-seeking cells on the level of single cell morphology, migration and molecular architecture are reflected by their transcriptomes.

## 4. Discussion

In this study, we explored the basic biological characteristics of osteotropic breast cancer cell lines. Our objective was to establish a potential relation between their specific tropism and their morpho-profiling, including the formation of specific membrane protrusions. Because these events are highly integrated multi-step processes, such as the molecular mechanisms regulating cell migration [60,61,62], we evaluated the architecture, migratory behaviors and biophysical properties of MB-231 cells and two enhanced bone-seeking sub-lines growing on a two-dimensional (2D) environment and correlated these findings with their phenotypic and molecular characteristics. To confine the specific osteotropic features, we analyzed a brain-seeking sub-line.

At the cellular level, we demonstrated that the heterogeneous population of MB-231 cells contains seven distinct and stable cell morphologies, which are independent from the choice of culture medium. These observations are in agreement with innovative studies on the evolution of cellular morphotypes in cancer metastasis [13,63], suggesting that the molecular traits underlying a given morphotype are heritable and propagate in the same way in individual cancer cell lines. This contrasts with the general idea that clones that grow faster than others are expected to dominate a cell population over time. These different morphotypes reflected a distinct motility pattern and their migration can be classified as a mesenchymal type in the case of morphotypes 3 to 6, and as amoeboid-like type in the case of Morphotype 7. Mesenchymal cell migration has been described for fibroblasts, invasive cancer cells and various developmental precursor states, particularly those observed during the epithelial–mesenchymal transition (EMT), whereas amoeboid cell migration has been observed for instance for hematopoietic cells and aggressive colon cancer cells [64,65,66,67]. Mesenchymal cell migration can be subdivided into two modes: discontinuous, as detected for Morphotypes 3, 4 and 6, and continuous, as exemplified for those with Morphotype 5 [68]. The majority of cells migrating in the discontinuous mode resulted in undirected cell movement. The lack of coordination between the front and rear poles is particularly evident in cells of Morphotypes 3 and 4. It also occurs, although to a lesser degree, in Morphotype 6, which shows a typical fibroblast-like mode of locomotion [69]. Whether this is the result of intrinsic or extrinsic cellular properties remains to be determined [70,71,72]. Indirectly, the morphotype shifting (i.e., Morphotypes 3 → 6 or 7 and 4 → 5) observed upon addition of EGF, especially in BONE cells, would suggest that the external signal regulated some of it. The shift from mesenchymal to amoeboid mode is called the mesenchymal–amoeboid transition, and may respond in vivo to a change in the cellular microenvironment [73]. By contrast, Morphotype 5 cells have less frequent changes in direction, resulting in a longer displacement distance. The EGF can also stimulate the formation of magnupodium (see below), and hence impact the migration of Morphotype 5 cells. This continuous mode of mesenchymal migration was described for the keratocytes where an outgrowth consistently led to a retraction, which enables cells to move in one direction [74]. It was proposed that cell–matrix adhesion complexes and actomyosin contractility may play a role in shaping the frequencies of discontinuous and continuous modes [68].

Among all morphologies, small rounded, highly motile cells, such as those described for Morphotype 7, stand out as they are reminiscent of Dictyostelium amoeba in which the corresponding migration is classified as an amoeboid type [75,76]. All MDA cells used in this study, however, did not produce substantial membrane blebs, as would be expected for this type of migration [77,78,79]. Instead, they bore a very large lamellipodium on the front edge—a similar phenotype has been described for Dictyostelium cell subjected to high osmolarity or deficient in myosin II [80]. Such a rounded phenotype has been previously observed for breast cancer cells [73,81,82] and the overall proportion of mesenchymal versus amoeboid migration types observed for parental MB-231 cells (this study) is in agreement with a previous report [73], suggesting the robustness of maintaining a given morphological and migratory heterogeneity in a cell line. Morphotype 7 is also similar to a subpopulation of hematopoietic stem cells and leukocytes, which could reach the bone marrow microenvironment and inflammation sites, respectively [24,83,84,85,86,87]. It is interesting to note that none of these rounded cells harbor an uropod-like structure at the rear pole, which is a specialized pseudopod backward projection that facilitate cell migration, notably the transendothelial migration [88]. The lack of a well-defined uropod could be explained, at least in part, by the 2D culture conditions, since others have shown that a premature uropod can be stimulated, to some degree, in a 3D environment [89,90].

Aside from the migrating cells, poorly motile cells are also of interest since they are differentially detected. Cells exhibiting Morphotype 2 harbor a magnupodium, as previously observed in a subpopulation of hematopoietic stem cells [24,91,92] and cancer cells [91,93]. In a previous study, one of our laboratories has shown that CD9 participates in their formation by stabilizing the lamellipodium at the free end [94], and it remains to be determined whether the differential CD9 expression explains the presence or absence of these types of protrusions in a given cell line. Magnupodia have also some similarity to long pseudopods and invadopodia that grow from the ventral part of the breast cancer cell cultured in a soft 3D environment [66,95,96]. It was demonstrated that EGFR-Src-Arg-cortactin pathway mediates functional maturation of invadopodia and breast cancer cell invasion [97], and the overexpression of the EGF receptor enhanced intravasation [98]. The increased amount of MB-231 and BONE cells with transient magnupodium upon the EGF incubation reinforces their relationship [99]. The presence of actin and the intermediate filament vimentin in invadopodia and magnupodia further strengthens this connection [96,100]. Other types of long and invasive protrusions, distinct to the invadopodia, have been described [41,101,102,103], and their relationship to magnupodium has yet to be determined. In the case of invadopodia, by providing the necessary proteinases, they stimulate the proteolytic degradation of the ECM network, which forms the basement membranes of epithelial and endothelial cells, and thus promote tumor cell invasiveness from the primary tumor [104,105,106].

Dissecting the morpho-profile in breast cancer could aid in predicting invasion, intravasation/extravasation and colonization capacities. For example, the reduced amounts of cells with magnupodia (Morphotype 2) and keratocyte-like feature (Morphotype 5) in the bone- and brain-seeking variants can be explained by their in vivo selection procedure in mice, since the experimental metastasis assay artificially introduces cancer cells into the bloodstream and ignores the metastatic invasion, migration and intravasation steps of the primary tumor. In line with this, markers of the EMT (e.g., E-cadherin, KRT8 and 19, SMA, TNC) are differentially expressed between the parental MB-231 cells and bone-seeking variants. The repetition of this selection process might also explain subset differences between MET and BONE cells [16]. Therefore, these morphotypes could be related to invasion and intravasation processes in primary sites. Although Morphotype 2 is rarer in bone-seeking variants, we could not exclude that a fraction of cells with magnupodium play a role in colonizing the bone marrow, as recently described for another type of long protrusions that make contact with osteogenic cells [41]. In contrast to MB-231 cells, both populations (MET and BONE) of bone-seeking cells are selectively enriched in the migrating cells of fibroblast-like Morphotype 6, and for the brain-seeking variant, in Morphotype 7. The latter might determine the trafficking of cancer cells inside the blood vessel during extravasation en route to bone marrow cavities among other targets. Interestingly, the stiffness of those harboring Morphotypes 5, 6 and 7, is lower compared to those with elongated fibroblastic Morphotypes 3 and 4, which is consistent with the potential implication of the former in intravasation and extravasation. In contrast, poorly motile cells with fibroblastic morphology, especially those of Morphotype 3, could play a role in the dissemination of breast cancer cells, e.g., in the stromal cell-compartment. It remains to be determined whether they are preferentially involved in the bone marrow colonization as proposed for hematopoietic stem cells [24,107]. Finally, it was surprising to find a significant proportion of cells, in both the parental and the brain-seeking variant, of the non-migrating Morphotype 1. Further investigations are needed to determine first the relation between this morphology and cellular function, and second, whether its reduced appearance is somehow related to the potential to metastasize to the bone, as observed with two variants with enhanced osteotropism. Altogether, establishing a relationship between cell morphology and the different steps of the metastatic process could help in understanding, and perhaps predicting, breast cancer metastasis [13,63].

At a molecular level, appropriate sets of proteins regulating the cell morphology and various steps of migration, including cell–ECM and cell–cell adhesions, and chemotaxis, are essential to determine the final destination of metastatic cancer cells [64]. Our analysis reveals a significant player regulating mesenchymal versus amoeboid migration: NEDD9 [108]. This protein localizes to focal adhesions and regulates their turnover. The elevated expression of NEDD9 is required for the mesenchymal migration mode as it could positively impact Rac1 GTPase and negatively the RhoA signaling pathways [58,109]. In an integrin β3-dependent mechanism, NEDD9 interacts with the guanine nucleotide exchange factor DOCK3 and promotes Rac1-WAVE2 activation, leading to an elongated, mesenchymal mode of migration [110,111]. This pathway could cause inhibition of the RhoA/ROCKII pathway, and thereby suppress the amoeboid movement through decreasing the actomyosin contractility. Conversely, in cells with an amoeboid movement, Rho-kinase signaling activates a Rac-GAP, ARHGAP22, which suppresses mesenchymal movement by inactivating Rac1. In addition to NEDD9, several other proteins in this molecular switch are differentially expressed, including integrin β3, ROCKII and ARHGAP22, which could explain the increased amount of rounded cells with amoeboid movement in the bone-seeking sub-lines. In such context, it is interesting to note that NEDD9 was highlighted as a positive regulator of EMT and promotes invasion in aggressive breast cancer cells such as MB-231 cells [112]. Furthermore, NEDD9 is crucial for the formation of invasive pseudopodia required for local matrix degradation at the cancer primary site [113], and thus may explain the presence of magnupodia in parental MB-231 cells.

Among the players involved in their navigation to metastatic sites and their cross-talk with resident cells [114,115], a differential expression of chemokine receptors (CXCR4, CXCR6, CCR1 and CCR7) was observed. The differential CXCR4 expression among the cell lines is consistent with its heterogeneous expression in parental MB-231 cells [116]. Its downregulation, notably in both bone-seeking sub-lines, was nevertheless surprising, since the CXCR4–CXCL12 pair plays a significant role in CD34^+^ hematopoietic stem cell homeostasis in the bone marrow microenvironment and in lymphocyte trafficking [117,118]. It was previously proposed that CXCR4 increases bone metastasis when overexpressed alone, but particularly in combination with IL11 and osteopontin, in MB-231 cells [119], suggesting that a functionally diverse set of genes cooperatively promote bone metastasis. As demonstrated in vivo with a neutralizing antibody, the CXCR4–CXCL12 pair may favor the lung and lymph node metastases [115,120]. A role in metastatic colonization of the liver was also documented [116]. Similarly, the downregulation of CCR7 in the bone-seeking variants could also be explained by its role and that of its ligands (CCL19 and CCL21) in lymph node metastasis [121,122,123]. Furthermore, both CXCR4 and CCR7 might regulate the signaling pathways that favor the pseudopodia formation [115], which is in line with the abundance or deficiency of magnupodia in MB-231 cells and bone- or brain-seeking variants, respectively (see above). The upregulation of CCR1 and CXCR6 and the downregulation of CXCR6 ligand (CXCL16) in cells other than MB-231, suggest that alternative chemokine-related axes may be involved in the trafficking of breast cancer cells to the bone marrow.

Beyond the regulation of the gene, other factors may influence the function of the protein, and thus its role in the metastasis process. For instance, the modification of glycosylation can alter many proteins, and thus change their functions and adhesive properties. In agreement with early studies [18,21], we observed differential expressions of glycosyltransferase genes between cell lines, suggesting that the glycomic phenotype of breast cancer cells may impact tissue-specific metastasis and secondary site colonization [55].

Altogether, our findings and those of others [13,119,124,125,126] provide a conceptual framework with a platform of phenotypic and molecular data that could help to dissect the characteristics of breast cancer cells, including their selective capacity for metastasis. The next step in our study will be to silence or overexpress the genes that are up- or downregulated, respectively, in the bone-seeking sub-lines, and to evaluate their potential in mouse models for promoting bone metastasis. The assignment of the given differentially expressed proteins to a given morphotype as well as the cell isolation of each morphotype and their in vivo transplantation could highlight their involvement in various aspects of the metastasis process, including intra- and extravasation. Besides the established cell lines, our analysis should be extended to primary breast cancer cells, which might provide a clinical relevance for predicting bone metastasis-free survival in breast cancer patients.

Similarly, it will be interesting, from a biological perspective, to study at a high resolution the characteristics of breast cancer cell lines growing on various supports other than fibronectin or co-cultured with bone marrow-derived stromal and/or osteogenic cells [83,127]. It is well established that the tissue/matrix stiffness could impact the morphology and migration of cancer cells [66]. Likewise, culturing them in a 3D scaffold might also be of interest particularly regarding the formation of magnupodium and uropod or other types of invasive protrusions [128]. Other cell lines, notably the luminal A (HR+/HER2-) subtype that preferably metastasizes to the bone, should be investigated as well. The impact of inflammatory cytokines that influence breast cancer bone metastasis should also be explored in the future.

## 5. Conclusions

We demonstrate that single cell morphology encodes the molecular, migratory and biophysical architecture of a breast cancer cell and is altered as soon as cells convert to an osteotropic phenotype. In this regard, quantitative morpho-profiling could aid in dissecting breast cancer heterogeneity and in refining the clinically relevant intrinsic subtypes of breast cancer in order to improve personalized treatment.

## Data Availability

The data presented in this study are available within the article/Supplementary Materials. Furthermore, we created a NCBI BioProject (PRJNA781764) and submitted our raw transcriptome data under the following accession numbers: SRR16979536 SRR16979530 SRR16979537 SRR16979538 SRR16979539 SRR16979529 SRR16979540 SRR16979533 SRR16979534 SRR16979531 SRR16979532 SRR16979535.

## Supplementary Materials

The following supporting information can be downloaded at: https://www.mdpi.com/article/10.3390/cancers14030603/s1, Figure S1: Expression of selected proteins in breast cancer cells; Figure S2: Differential expression of integrins between breast cancer cell lines; Figure S3: Differential expression of cytoplasmic proteins and their transcripts between breast cancer cell lines; Figure S4: Binding of lectins to breast cancer cell lines; Figure S5: Differential gene expression in bone-seeking variants; Figure S6: Differential gene expression in the brain-seeking variant; Figure S7: Differential gene expression in bone-seeking variants associated with different pathways; Figure S8: Differential gene expression in bone-seeking variants associated with different protein families. Videos S1–S11: These videos depict breast cancer cell lines without and with motility; Videos S12–S15: These videos depict breast cancer cell lines during the cell division; Videos S16 and S17: These videos depict breast cancer cell lines upon incubation with EGF. Table S1: List of primary antibodies; Table S2: List of secondary antibodies.
