# Supplementary material for: Decoding Single Cell Morphology in Osteotropic Breast Cancer Cells for Dissecting Their Migratory, Molecular and Biophysical Heterogeneity
Source: Cancers (Basel). 2022 Jan 25;14(3):603. doi: 10.3390/cancers14030603 (PMC8833404; doi:10.3390/cancers14030603)
Supplement: Supplementary file 1 [file cancers-14-00603-s001.zip › cancers-1504450_supplementary material.pdf]

cancers

Supplementary Materials

Decoding Single Cell Morphology in Osteotropic Breast Cancer Cells for Dissecting Their Migratory, Molecular and Biophysical Heterogeneity

Lila Bemmerlein , Ilker A. Deniz, Jana Karbanová, Angela Jacobi, Stephan Drukewitz, Theresa Link, Andy Göbel, Lisa Sevenich, Anna V. Taubenberger, Pauline Wimberger, Jan Dominik Kuhlmann and Denis Corbeil

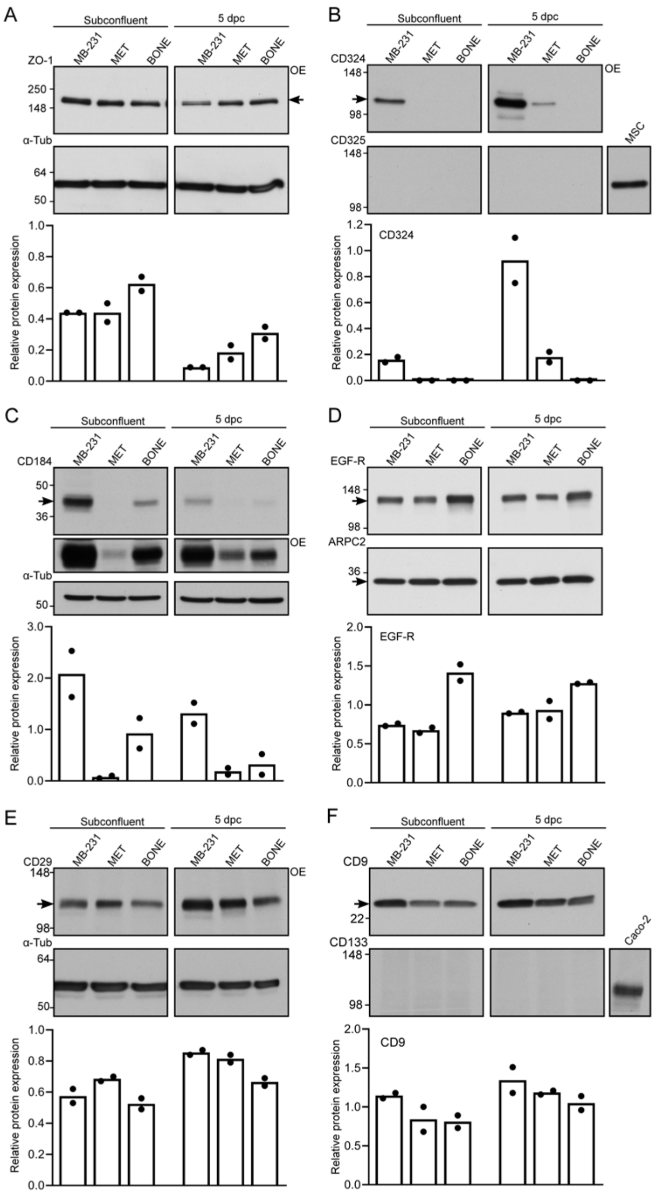

Figure S1. Expression of selected proteins in breast cancer cells. **A–F** Detergent cell lysates prepared from subconfluent and 5 days postconfluent (5 dpc) MB-231, MET and BONE cells were separated by SDS-PAGE under non-reducing (for CD9) or reducing (for others) conditions, and analyzed by immunoblotting using specific antibodies against selected proteins as indicated. Human mesenchymal stromal cells (MSC) [1] and colon carcinoma Caco-2 cells [2] were used as positive controls. Molecular mass markers (kDa) are shown. Arrows indicate proteins of interest. In

some cases, blots were overexposed (OE, i.e. 20 min instead 30 sec) to highlight the low expression of certain proteins. The relative expression of a given protein was quantified and normalized to α-tubulin as a housekeeping protein. The mean of 2 independent experiments is presented, and each point represents the value of the individual experiment. EGF-R, EGF receptor.

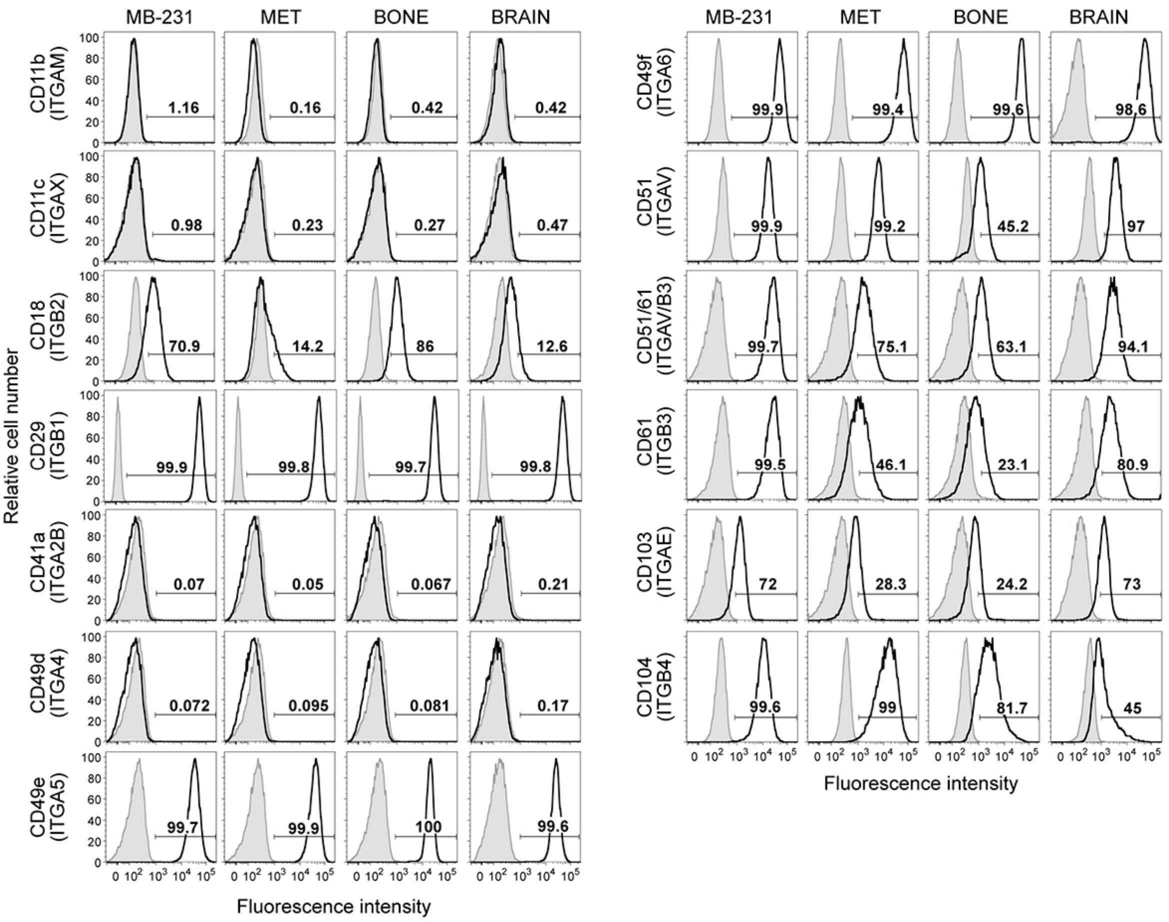

Figure S2. Differential expression of integrins between breast cancer cell lines. The parental cells MB-231 and its bone- and brain-seeking derivatives, MET/BONE and BRAIN, respectively, were cell surface immunolabeled for integrin molecules as indicated prior to flow cytometry analyses. Percentages of positive cells are indicated in the histograms. A representative experiment is displayed.

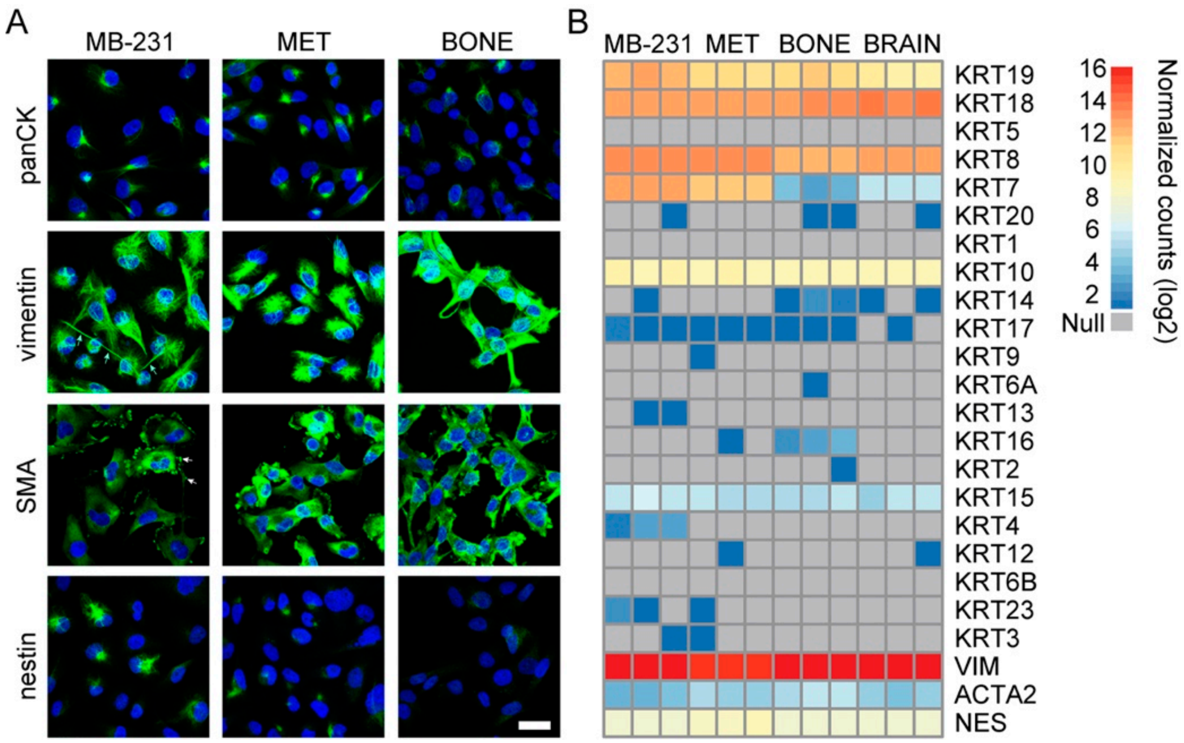

Figure S3. Differential expression of cytoplasmic proteins and their transcripts between breast cancer cell lines. **A** The parental cells MB-231 and its bone-seeking variants, MET and BONE, were fixed, permeabilized and immunolabeled for one of the indicated proteins, as indicated prior to cytochemistry. **B** Heatmap representations of differentially expressed genes. Note the enrichment of vimentin (VIM) and alpha smooth muscle actin (SMA, ACTA2) along the magnupodium (green arrow) or at its extremity (white arrow), respectively, in MB-231 cells (**A**). CK, cytokeratin. Scale bar, 25 µm.

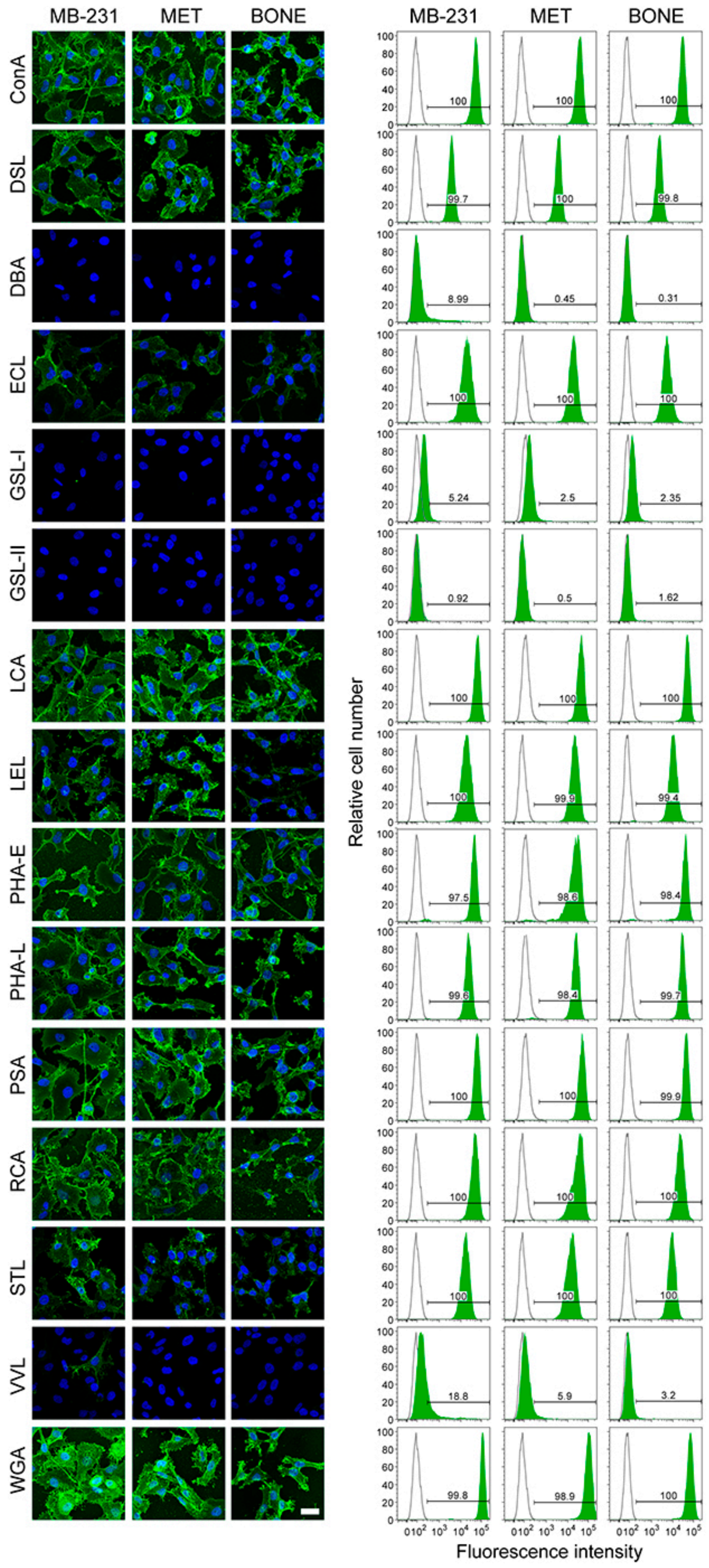

Figure S4. Binding of lectins to breast cancer cell lines. The parental cells MB-231 and its bone-seeking derivatives, MET and BONE, were cell surface labeled with one of a panel of distinct FITC-conjugated lectins prior to cytochemistry (left panels) and flow cytometry (right panels).

Numbers of positive cells are indicated in the histograms. Representative experiments are displayed. Scale bar, 25 µm.

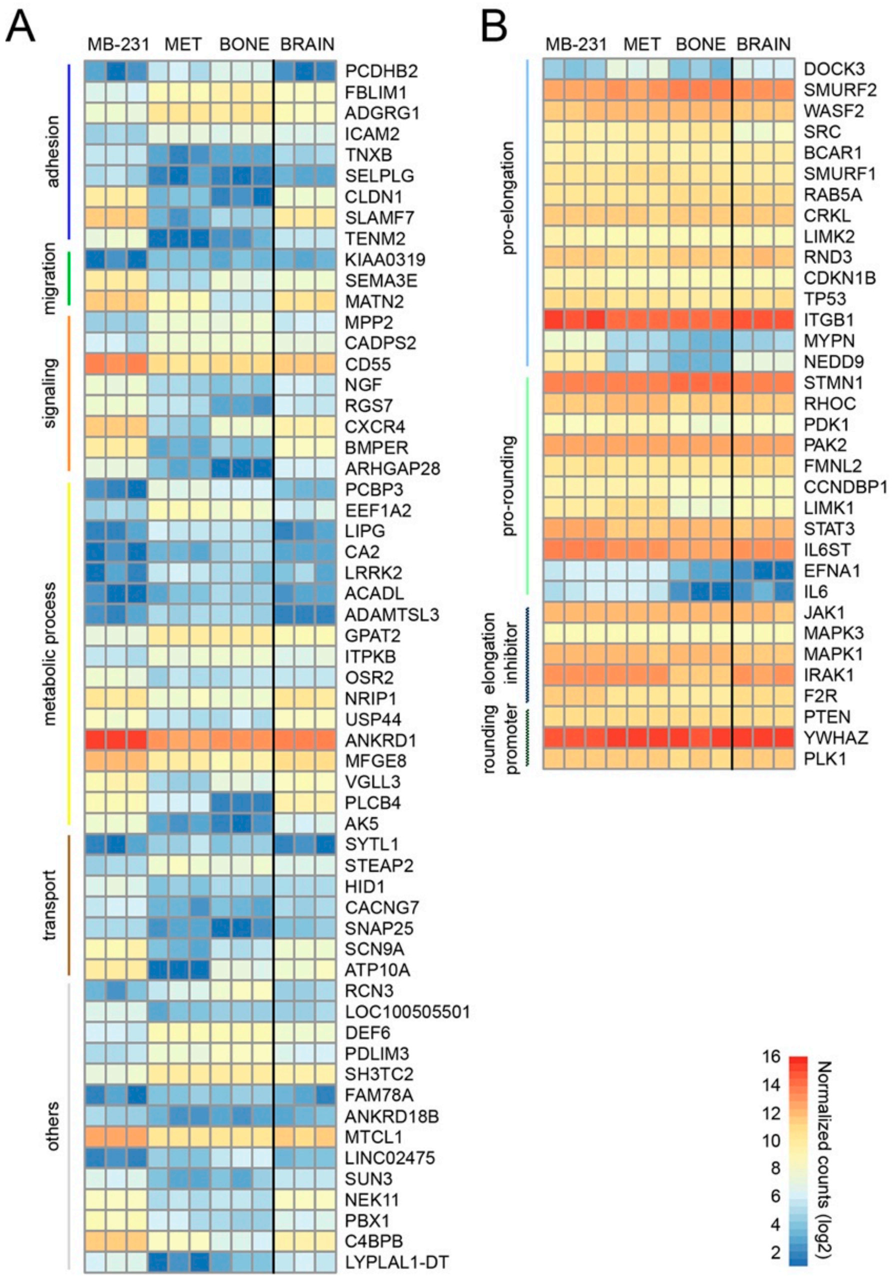

Figure S5. Differential gene expression in bone-seeking variants. **A, B** The heatmaps of genes (58) that are significantly differentially expressed (log2 fold change >2 or <−2) in the bone-seeking variants (MET and BONE), but not in the brain-seeking variant (BRAIN), relative to the parental cells MB-231 (**A**, see Figure 9D) and those with impact on cell shape (**B**, see Figure 9G) are presented.

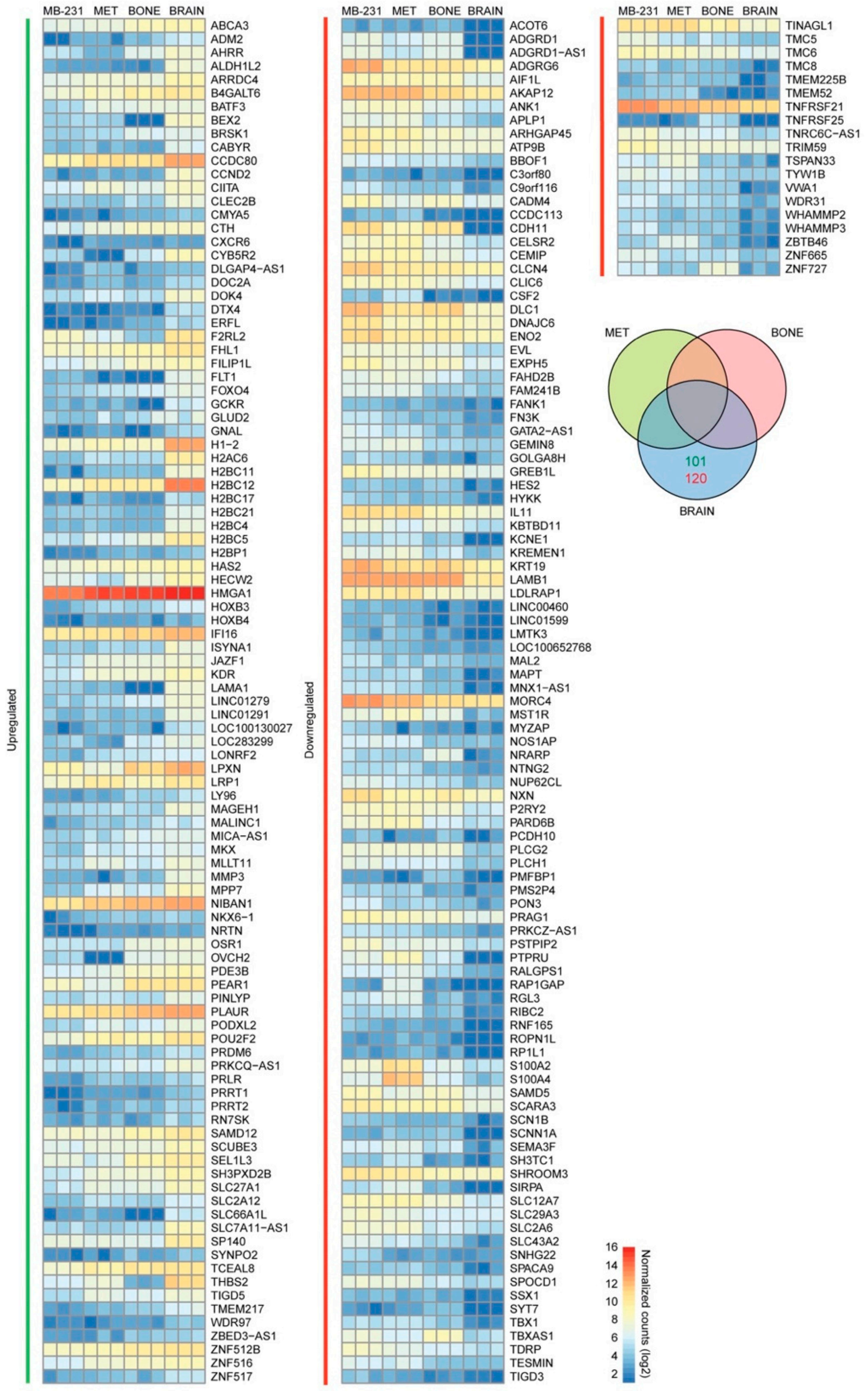

Figure S6. Differential gene expression in the brain-seeking variant. The heatmap and Venn diagram of the genes that are significantly differentially expressed only in the brain-seeking variant (BRAIN), but not in bone-seeking variants (MET and BONE), relative to the parental cells MB-231

cells are presented. Genes that are up- (101) and down- (120) regulated (log2 fold change >2 or <−2, respectively) are shown in green and red, respectively.

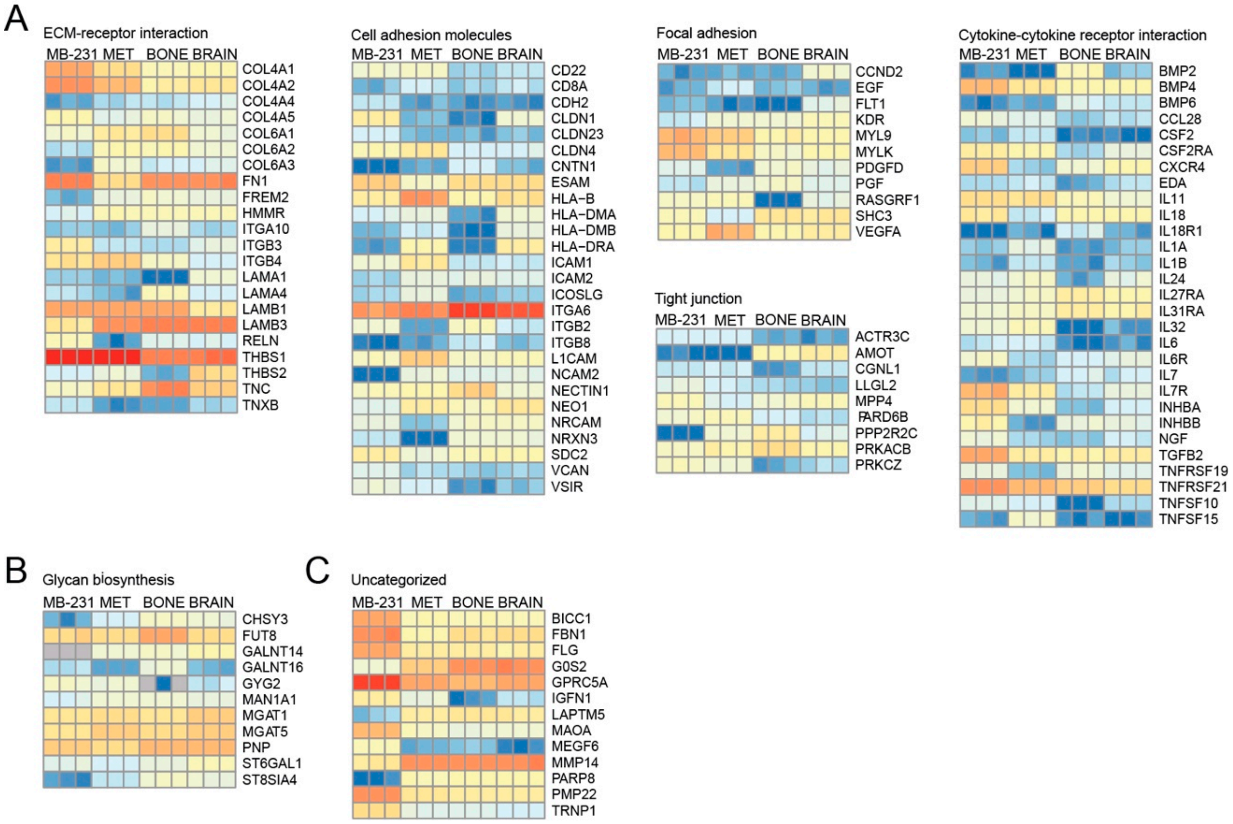

Figure S7. Differential gene expression in bone-seeking variants associated with different pathways. **A–C** Heatmap representations of significantly differentially expressed genes between the bone-seeking variants (MET and BONE), brain-seeking variant (BRAIN), and the parental cell line MB-231 (log2 fold change >2 or <−2). The genes are classified into distinct KEGG pathways as indicated.

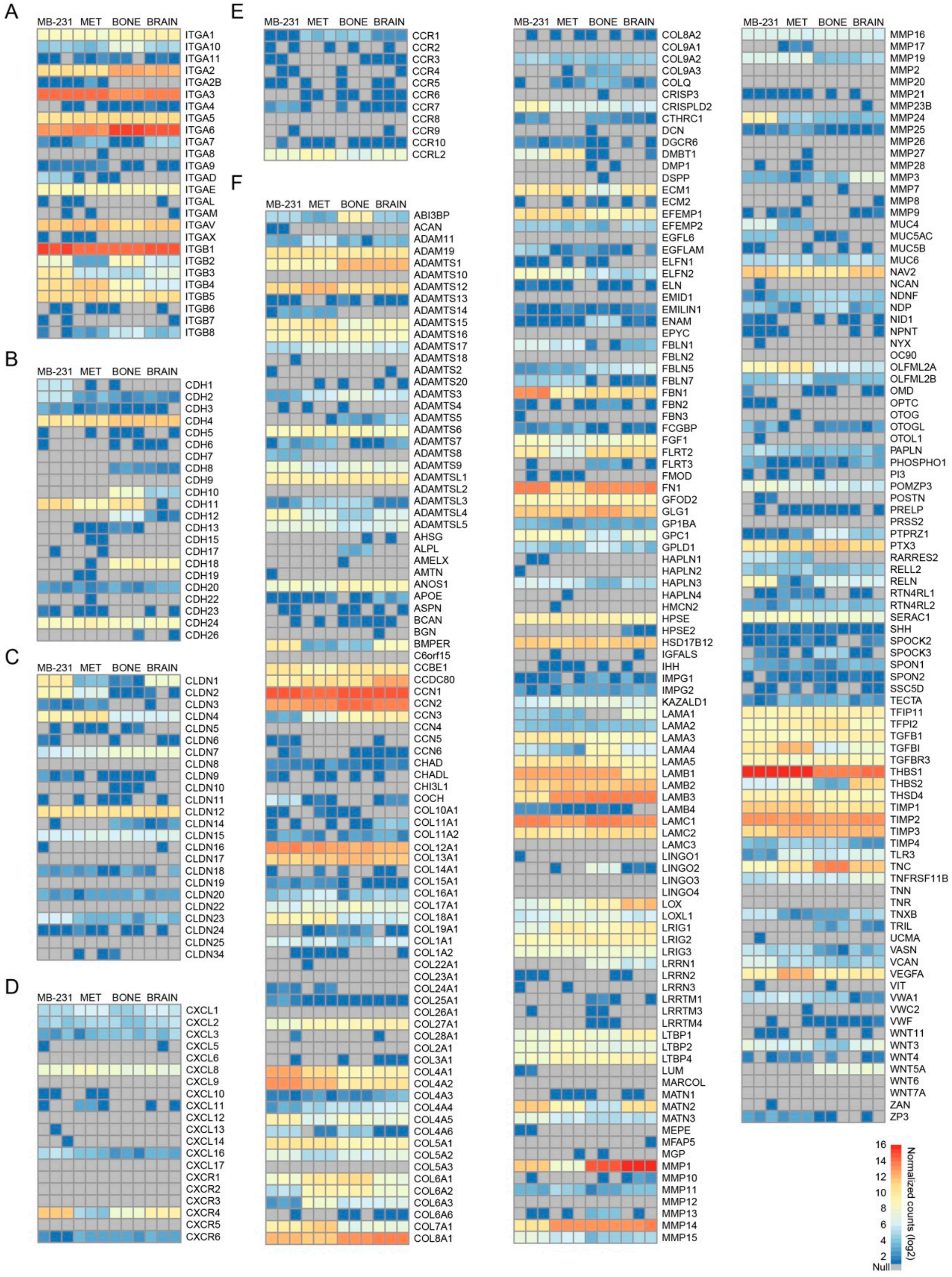

Figure S8. Differential gene expression in bone-seeking variants associated with different protein families. **A–F** Heatmaps of genes that are belonging to integrin (**A**), cadherin (**B**), claudin (**C**) protein families, chemokines and their receptors (**D**, **E**) and extracellular-matrix components (**F**, gene ontology list GO:0031012) are shown without prior filtering.

Supplementary Videos

Video S1–S11. These videos depict breast cancer cell lines, as indicated in the name file, without and with motility. The elapsed time is shown in the right corner. Still images from these movies are shown in Figure 2 (B, Videos S1-S6); D, Videos S7-S11). (Format: mov; size: <1 MB).

Video S12–S15. These videos depict breast cancer cell lines, as indicated in the name file, during the cell division. The elapsed time is shown in the right corner. Still images from these movies are shown in Figure 3B. (Format: mov; size: <2 MB).

Video S16–S17. These videos depict breast cancer cell lines, as indicated in the name file, upon incubation with EGF. The elapsed time is shown in the right corner. Still images from these movies are shown in Figure 4 (E, right panels, Videos S16; F, Videos S7). (Format: mov; size: <1.5 MB).

Table S1. List of primary antibodies.

Primary antibody	Clone	Manufacturer[1]	Dilution		
			WB	FC	ICC/ IHC
α-tubulin	DM1A	Sigma-Aldrich	1:2500		
α-SMA	1A4	Sigma-Aldrich			1:200
ARPC2[2]	EPR8533	Abcam	1:1000		
CD9	M-L13	BD Biosciences	1:1000		
CD9-PE	HI9a	BioLegend		1:20	
CD11b-PE	M1/70.15.11.5	Miltenyi Biotec		1:10	
CD11c-APC	B-ly6	BD Biosciences		1:5	
CD18	6.7	BD Biosciences		1:50	
CD29	12G10	Abcam	1:500		
CD29-APC	TS2/16	BioLegend		1:20	
CD41a-APC	HIP8	BD Biosciences		1:5	
CD44-PE	G44-26	BD Biosciences		1:5	
CD49d-APC	MZ18-24A9	Miltenyi Biotec		1:10	
CD49e-APC	NKI-SAM-1	BioLegend		1:20	
CD49f	GoH3	BD Biosciences		1:50	
CD51-PE	NKI-M9	BioLegend		1:20	
CD51/61-APC	23C6	BioLegend		1:20	
CD54	HA58	BD Biosciences			1:50
CD54-PE	HA58	BD Biosciences		1:5	
CD57	HNK-1	Thermo Fisher Sc.		1:20	
CD61-APC	VI-PL2	BioLegend		1:20	
CD62L-APC	DREG-56	BioLegend		1:20	
CD63-PE	CLB-gran/12, 435	Sanquin		1:10	
CD73-PE	AD2	BD Biosciences		1:5	
CD81	M38	Exbio		1:100	1:100
CD87-PE	VIM5	BioLegend		1:20	
CD90	5E10	BD Biosciences		1:50	
CD102-PE	CBR-1C2/2	BD Biosciences		1:5	
CD103-APC	B-ly7	Thermo Fisher Sc.		1:5	
CD104-PE	58XB4	BioLegend		1:20	
CD105	43A3	BioLegend			1:50
CD105-PE	SN6	Thermo Fisher Sc.		1:5	
CD117-APC	104D2	BioLegend		1:20	
CD133-APC	AC133	Miltenyi Biotec		1:10	
CD133	80B258	Karbanová et al. [3]	1:1667		
CD146-PE	P1H12	BD Biosciences		1:5	
CD162-PE	KPL-1	BioLegend		1:5	
CD166-PE	3A6	BD Biosciences		1:5	
CD184[2]	UMB2	Abcam	1:500		
CD184	12G5	BD Biosciences		1:50	1:50
CD271-APC	ME20.4-1.M4	Miltenyi Biotec		1:10	
CD309-APC	7D4-6	BioLegend		1:20	
CD324	36/E-Cadherin	BD Biosciences	1:5000		
CD325	32/N-Cadherin	BD Biosciences	1:1000		

c-erbB2 (HER-2)[2]	as	Dako			1:600
Estrogen receptor	SP1	Ventana/Roche			ready to use
EGF R[2]	D38B1	Cell Signaling Tech.	1:1000		
EGF R-APC	AY13	BioLegend		1:20	
FGF R2-APC	98725	Neuromics		1:5	
IgG1 isotype control–PE, APC	MOPC-21	BD Biosciences		1:5	
IgG2a isotype control–PE	MOPC-173	BioLegend		1:20	
IgG2b isotype control-PE, APC	IS6-11E5.11	Miltenyi Biotec		1:10	
Ki67	MIB-1	Dako			1:50
MFG-E8	278918	R&D systems		1:25	1:25
Nestin	10C2	Chemicon			1:100
NG2-PE (CSPG4)	7.1	Beckman Coulter GmbH		1:5	
PanCK	C11	Santa Cruz Biotech.			1:33
Progesterone receptor[2]	1E2	Roche			ready to use
Vimentin	V9	Santa Cruz Biotech.			1:33
ZO-1[2]	D6L1E	Cell Signaling Tech.	1:1000		1:400

[1]Abcam (Cambridge, UK); BD Biosciences (Heidelberg, Germany); Beckman Coulter GmbH (Krefeld, Germany); BioLegend (San Diego, CA, USA); Cell Signaling Technology (Danvers, MA, USA); Dako (Santa Clara, CA, USA), Exbio (Prague, Czech Republic); Chemicon (Temecula, CA, USA); Miltenyi Biotec (Bergisch Gladbach, Germany); Neuromics (Edina, MN, USA); R&D systems (Minneapolis, MN, USA); Sanquin (Amsterdam, The Netherlands); Santa Cruz Biotechnology, Inc. (Dallas, TX, USA); Sigma Aldrich (Darmstadt, Germany); Thermo Fisher Scientific (Waltham, MA, USA); Ventana/Roche (Mannheim, Germany).
[2]Antibody generated in rabbit.
As, antiserum; FC, flow cytometry; ICC, immunocytochemistry; IHC, immunohistochemistry; APC, allophycocyanin; PE, phycoerythrin; WB, immunoblotting.

Table S2. List of secondary antibodies.

**Secondary antibody**	**Manufacturer**[1]	**Dilution**		
		**WB**	**FC**	**ICC/ IHC**
Alexa Fluor™ 488–conjugated goat anti-mouse IgG1 specific (H+L)	Thermo Fisher Scientific			1:600
Alexa Fluor™ 488–conjugated goat anti-mouse IgG2a specific (H+L)	Thermo Fisher Scientific			1:600
Alexa Fluor™ 488–conjugated goat anti-rabbit IgG (H+L)	Thermo Fisher Scientific			1:600
Alexa Fluor™ 633–conjugated goat anti-mouse IgM specific (H+L)	Thermo Fisher Scientific		1:600	
APC-conjugated F(ab')2 goat anti-mouse	Thermo Fisher Scientific		1:100	
PE-conjugated F(ab')2 donkey anti-rat IgG	Thermo Fisher Scientific		1:100	
PE-conjugated F(ab')2 goat anti-mouse	Thermo Fisher Scientific		1:100	
HRP-conjugated AffiniPure goat anti-mouse IgG	Jackson ImmunoResearch	1:3000		
HRP-conjugated AffiniPure goat anti-rabbit IgG	Jackson ImmunoResearch	1:3000		

[1]Thermo Fisher Scientific (Waltham, MA, USA), Jackson ImmunoResearch Europe Ltd. (Ely, UK). APC, allophycocyanin; FC, flow cytometry; HRP, horseradish peroxidase; ICC, immunocytochemistry; IHC, immunohistochemistry; PE, phycoerythrin; WB, immunoblotting.

Supplementary References

1. Freund, D.; Fonseca, A.V.; Janich, P.; Bornhäuser, M.; Corbeil, D. Differential expression of biofunctional GM1 and GM3 gangliosides within the plastic-adherent multipotent mesenchymal stromal cell population. *Cytotherapy* **2010,** *12*, 131–142, doi:10.3109/14653240903476438.
2. Corbeil, D.; Röper, K.; Hellwig, A.; Tavian, M.; Miraglia, S.; Watt, S.M.; Simmons P.J.; Peault, B.; Buck, D.W.; Huttner W.B. The human AC133 hematopoietic stem cell antigen is also expressed in epithelial cells and targeted to plasma membrane protrusions. *J. Biol. Chem.* **2000,** *275,* 5512–5520, doi:10.1074/jbc.275.8.5512.
3. Karbanová, J.; Missol-Kolka, E.; Fonseca, A.V.; Lorra, C.; Janich, P.; et al. The stem cell marker CD133 (Prominin-1) is expressed in various human glandular epithelia. *J. Histochem. Cytochem.* **2008,** *56*, 977–993, doi:10.1369/jhc.2008.951897.
