# Supplementary figures and images for: Decoding Single Cell Morphology in Osteotropic Breast Cancer Cells for Dissecting Their Migratory, Molecular and Biophysical Heterogeneity
Source: Cancers (Basel). 2022 Jan 25;14(3):603. doi: 10.3390/cancers14030603 (PMC8833404; doi:10.3390/cancers14030603)

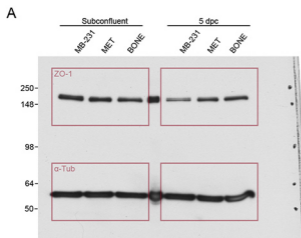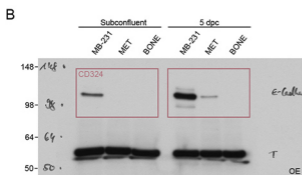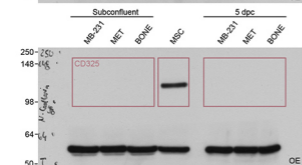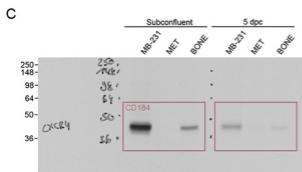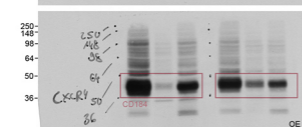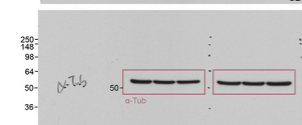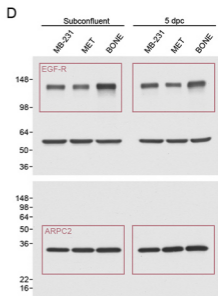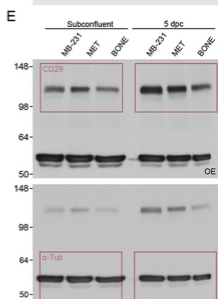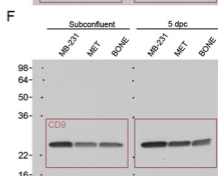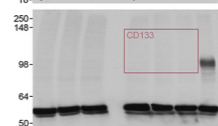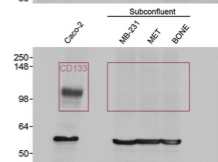

Supplement: Supplementary file 1 [file cancers-14-00603-s001.zip › cancers-1504450_uncropped_WB.pdf]
